# Long Non-Coding RNAs in Pancreatic Cancer: Biologic Functions, Mechanisms, and Clinical Significance

**DOI:** 10.3390/cancers14092115

**Published:** 2022-04-24

**Authors:** Jiajia Li, Sicong Hou, Ziping Ye, Wujun Wang, Xiaolin Hu, Qinglei Hang

**Affiliations:** 1 Department of Gastroenterology, The Affiliated Hospital of Yangzhou University, Yangzhou 225009, China; nylijiajia@126.com (J.L.); shou@yzu.edu.cn (S.H.); 2 Department of Clinical Medicine, Medical College, Yangzhou University, Yangzhou 225001, China; hxl15722563995@163.com; 3 Department of Gastroenterology, The First Affiliated Hospital of Nanjing Medical University, Nanjing 210029, China; yzp1998@126.com; 4 Nanjing Hospital of Chinese Medicine Affiliated to Nanjing University of Chinese Medicine, Nanjing 210023, China; wangwujunnzy@outlook.com; 5 Department of Experimental Radiation Oncology, The University of Texas MD Anderson Cancer Center, Houston, TX 77030, USA

**Keywords:** LncRNAs, pancreatic cancer, biomarker, cancer diagnosis and therapy

## Abstract

**Simple Summary:**

Pancreatic cancer (PC) is a highly aggressive malignant tumor with a high mortality rate. Growing evidence shows that long non-coding RNAs (lncRNAs) might participate in the pathogenesis of PC. This review presents the biogenesis mechanism, classifications, and modes of action of lncRNAs, especially the functions and mechanisms of lncRNAs in PC. It also discusses the clinical significance of lncRNAs in PC.

**Abstract:**

Despite tremendous efforts devoted to research in pancreatic cancer (PC), the mechanism underlying the tumorigenesis and progression of PC is still not completely clear. Additionally, ideal biomarkers and satisfactory therapeutic strategies for clinical application in PC are still lacking. Accumulating evidence suggests that long non-coding RNAs (lncRNAs) might participate in the pathogenesis of diverse cancers, including PC. The abnormal expression of lncRNAs in PC is considered a vital factor during tumorigenesis that affects tumor cell proliferation, migration, invasion, apoptosis, angiogenesis, and drug resistance. With this review of relevant articles published in recent years, we aimed to summarize the biogenesis mechanism, classifications, and modes of action of lncRNAs and to review the functions and mechanisms of lncRNAs in PC. Additionally, the clinical significance of lncRNAs in PC was discussed. Finally, we pointed out the questions remaining from recent studies and anticipated that further investigations would address these gaps in knowledge in this field.

## 1. Introduction

Pancreatic cancer (PC) is the fourth leading cause of cancer-related death in the United States and ranks seventh in terms of cancer-related death worldwide. Globally, nearly 5 million new cases are diagnosed each year, almost equal to the number of deaths caused by PC, and the five-year survival rate is approximately 10% [1,2]. Currently, surgical resection is the only curative treatment option available for PC patients [3]. However, only 10–20% of patients have the option of surgery when diagnosed, and the 5-year survival rate remains relatively low [4]. Although efforts in recent years have partially improved the efficacy of surgery and chemoradiotherapy, as exemplified by the application of the adjuvant chemotherapy regimens referred to as Folfirinox, the overall prognosis of PC patients is still unoptimistic. The key to improving the prognosis of PC is early diagnosis and precision treatment, making it critical to find ideal biological biomarkers for PC. Unfortunately, there is still a lack of credible biomarkers and effective therapeutic strategies for clinical application in PC [5].

In recent years, genetic and transcriptional sequencing have revealed the extensive heterogeneity of cancers. Many studies target molecular substrates such as long non-coding RNAs (lncRNAs) as biomarkers for early diagnosis and targeted therapy strategies for tumors. Despite the countless barriers, great progress has been made. LncRNAs are a type of noncoding RNAs (ncRNAs) with a size from 200 to more than one hundred thousand nucleotides [6]. Accounting for the majority (80 to 90%) of all ncRNAs with limited or no protein-coding properties, they are mainly responsible for gene regulation and are implicated in a plethora of biological processes [7]. The abnormal expression of lncRNAs in various cancers has been considered a vital factor during tumorigenesis that affects tumor cell proliferation, migration, invasion, apoptosis, angiogenesis drug resistance, etc. [8,9]. Recently, many studies have reported the important role of lncRNAs in the carcinogenesis of PC. Herein, we reviewed relevant articles published in recent years to summarize the biogenesis mechanisms, classifications, and modes of action of lncRNAs and review the functions and mechanisms of lncRNAs in PC. Additionally, we highlighted the questions remaining from recent studies and anticipate that further investigations will address these gaps in knowledge in this field.

## 2. Biogenesis and Localization of LncRNAs

Similar to mRNAs, the transcription of most lncRNA species is dependent on RNA polymerase II (Pol II) and involves the addition of 5′-end m7G caps and 3′-end poly(A) tails. However, compared with mRNAs, many Pol II-transcribed lncRNAs are inefficiently processed and retained in the nucleus [10,11,12]. Briefly, the mechanism of lncRNA retention in the nucleus can be summarized as follows: (a) Some lncRNAs are transcribed by an aberrant phosphorylated form of Pol II, which leads to splicing defects and polyadenylation signal-independent transcription termination during lncRNA transcription. These lncRNAs tend to be tethered to chromatin and are often degraded by nuclear exosomes [13]. (b) Many lncRNAs contain U1 small nuclear RNA (U1 snRNA) binding sites, which assist in the recruitment of U1 small nuclear ribonucleoprotein (U1 snRNP) to Pol II. This recruitment could further enhance the association of lncRNAs with chromatin [14]. (c) The splicing signals in some lncRNAs are relatively weak, and the distance between the 3′ splice site and the branch point is long. These features endow these lncRNAs with relatively low splicing activity and support their nuclear retention [12,15,16,17]. (d) Specific sequence motifs in cis and factors in trans could lead to nuclear retention [18,19,20]. In summary, the localization of lncRNAs in the nucleus is sophisticatedly controlled from the transcription level to the RNA processing level and involves the cooperation of diverse sequence motifs in cis and factors in trans. Nevertheless, despite the numerous patterns discussed above, more investigations into the underlying mechanism that determines the different retention modes of lncRNAs in the nucleus are needed.

While some lncRNAs are retained in the nucleus, a large number of lncRNAs are exported to and localized in the cytosol. Due to the limited number of exons contained in the sequences, the export of lncRNAs mainly relies on the nuclear RNA export factor 1 (NXF1) pathway [21]. After reaching the cytoplasm, lncRNAs can exist in diverse forms and interact with various RNA binding proteins (RBPs) in the cytoplasm, associate with ribosomes, and associate with the mitochondria [22,23,24,25,26]. A majority of cytoplasmic lncRNAs are reported to associate with ribosomes. This process has been further proved to be partially dependent on cis-elements such as long ‘pseudo’ 5′ untranslated regions. However, whether the lncRNAs found in polysome fractions take part in translation is unknown [23]. Apart from ribosomes, the mitochondria are also a destination of cytoplasmic lncRNAs. Many mitochondrial lncRNAs exist in the form of mitochondrial RNA-processing endoribonuclease (RMRP) and bind with G-rich RNA sequence-binding factor 1 (GRSF1), which facilitates the further accumulation of lncRNAs in the mitochondria [26]. Emerging evidence has identified many lncRNAs in other organelles, especially exosomes. It remains unclear how lncRNAs are guided to exosomes, but the possible mechanism might involve them binding with RBPs via specific sequence motifs [27,28,29].Given their tremendous diagnostic and predictive potential in various clinical settings, more in-depth research into the localization mechanism of lncRNAs in exosomes is imperative.

## 3. Classifications of LncRNAs

According to different characteristics, lncRNAs can be sorted into corresponding types: (a) Based on genome location, lncRNAs can be divided into intergenic lncRNAs, intronic lncRNAs, and exonic lncRNAs. Intergenic lncRNAs refer to lncRNAs transcribed from genomic regions between coding genes; intronic lncRNAs derive totally from introns, while exonic lncRNAs share some sequences with exons [30,31]. (b) Compared with protein-coding genes, lncRNAs can be classified as sense or antisense lncRNAs according to the transcriptional orientation [32]. (c) According to the subcellular localization, lncRNAs can be categorized as nuclear or cytoplasmic, categories which are closely related to the mechanism by which they exert their biological functions [33]. (d) Concerning the mode of action, lncRNAs can function in cis or in trans, which depends on the relative position of lncRNAs and the target genes [30,34] (Table 1).

## 4. Roles of LncRNAs in PC

**Figure 1:**
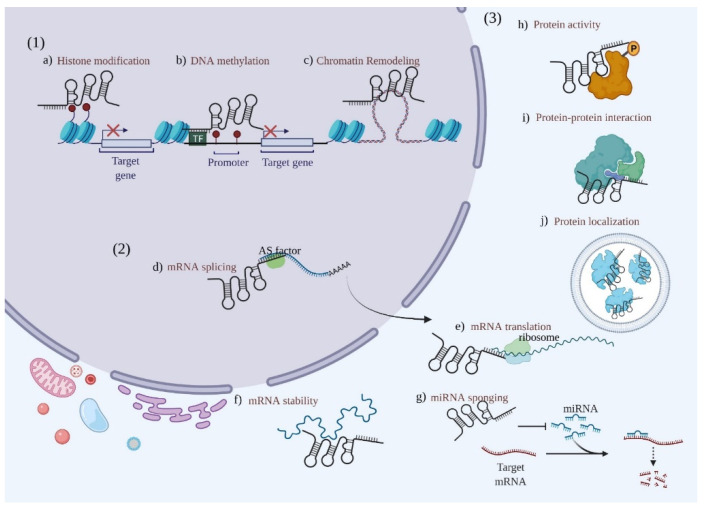
Mechanisms underlying long noncoding RNA (lncRNA)-mediated regulation of gene expression. (**1**) Transcription regulation by lncRNAs. (**a**) LncRNAs can promote histone H3 Lys27 trimethylation (H3K27me3) of the promoter region of chromatin via the recruitment of histone complexes such as polycomb repressive complex 2 (PRC2), thereby leading to the silencing of gene transcription; (**b**) lncRNAs can also catalyze DNA methylation to modulate transcriptive activity; (**c**) lncRNAs can enhance the expression of protein coding genes (PCGs) by remodeling chromatin (for example, by forming chromatin loops). (**2**) LncRNAs interact with RNAs. (**d**) Together with alternative splicing factors, lncRNAs are engaged in the processing and maturation of mRNAs; (**e**) lncRNAs facilitate mRNA translation; (**f**) lncRNAs help to stabilize some mRNAs by binding to them; (**g**) lncRNAs can competitively bind to miRNAs by acting as ceRNAs, thereby blocking the inhibition of the target gene. (**3**) LncRNAs interact with proteins. (**h**) Through protein modulation (for example, phosphorylation), lncRNAs regulate protein activity; (**i**) lncRNAs mediate the interactions between proteins; (**j**) lncRNAs regulate the localization of proteins.

LncRNAs delicately regulate gene expression at multiple levels. LncRNAs interact with DNA and proteins to regulate diverse biological processes, including histone modification, DNA methylation, hydroxymethylation, and chromatin remodeling. In contrast, the interaction of lncRNAs with RNAs (including miRNAs) could regulate RNA splicing, stability maintenance, and translation, thereby exerting a posttranscriptional modulation function [35,36]. In addition, lncRNAs can contribute to regulating protein activity, stability, and protein–protein interactions through diverse interactions with proteins [37] (Figure 1). Hereafter, we reviewed the roles of lncRNAs in PC tumorigenesis and progression and highlighted the involved molecular mechanisms (Table 2 and Table 3 and Tables S1 and S2).

### 4.1. LncRNAs Act as Histone Modulators

Numerous studies have reported that lncRNAs could facilitate histone modification in PC, thereby engaging in regulating the expression of target genes. In this process, polycomb repressive complex 2 (PRC2), consisting of an enhancer of zeste homolog 2 (EZH2), a suppressor of zeste 12 homolog (SUZ12), and embryonic ectoderm development (EED), acts as a vital mediator [38,39]. By forming complexes with various lncRNAs, PRC2 could enhance histone H3 Lys27 trimethylation (H3K27me3) to repress the expression of various genes [40,41]. The role of PRC2 components, especially EZH2, has been proven in a wide range of tumors, including breast cancer, prostate cancer, and lung cancer [40,42,43]. The interaction of PRC2 with lncRNAs in PC has also been reported by many investigators.

**Figure 2:**
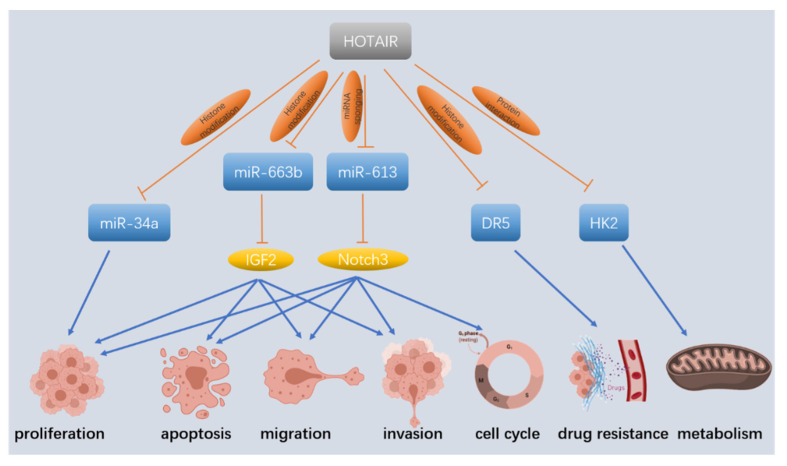
LncRNA Hox transcript antisense RNA (HOTAIR) regulates PC development in diverse pathways. LncRNA HOTAIR acts through histone modification, miRNA sponging, and protein interaction to regulate proliferation, apoptosis, migration, invasion, the cell cycle, drug resistance, and cell metabolism in PC. IGF2: insulin-like growth factor 2; DR5: death receptor 5; hexokinase-2 (HK2).

LncRNA Hox transcript antisense RNA (HOTAIR) is a negative prognostic factor for overall survival (OS) in patients with breast and colon cancer, and its high expression is implicated in the metastasis of breast and colon cancer [44,45,46,47]. Similar results were found by Kim et al. in PC. Gene array studies revealed the minimal overlap of genes regulated by HOTAIR between PC and breast cancer cells, and further research using EZH2 knockdown and chromatin immunoprecipitation demonstrated that gene repression mediated by HOTAIR was both PRC2-dependent and PRC2-independent [48]. Several miRNAs were reported to be regulated by HOTAIR through interaction with PRC2. In PC, Cai et al. found that HOTAIR suppressed the expression of miR-663b via the H3K4me3 and H3K27me3 histone modification of the miR-663b promoter. This epigenetic modification-mediated decrease in miR-663b further led to upregulation of its target insulin-like growth factor 2 (IGF2), a previously verified oncogenic factor in PC [49,50,51]. Another miRNA, miR-34a, was also reported to be regulated by HOTAIR via histone modification. In this study, the overexpression of EZH2 in human pancreatic ductal epithelial (HPDE) cells repressed miR-34a expression. Mechanistically, HOTAIR physically interacted with EZH2, which induced the occupancy of EZH2 at the miR-34a promoter, thereby repressing miR-34a transcription through the induction of heterochromatin formation [52]. In addition to the miRNAs which are transcriptionally dependent on EZH2, HOTAIR also participates in regulating many protein-coding genes. In PANC-1 and AsPC-1 cells, HOTAIR expression can be induced by radiation and its knockdown restored radiosensitivity by modulating ATG7-mediated autophagy [53]. In addition, by stimulating hexokinase-2 (HK2) expression, HOTAIR may be engaged in the processes of cancer cell energy metabolism, including glucose uptake, lactate production, and ATP production [54] (Figure 2).

LncRNA HOXA distal transcript antisense RNA (HOTTIP) is a HOX-associated lncRNA transcribed from the 5′ tip of the HOXA locus. By interacting with PRC2 and WD repeat-containing protein 5 (WDR5)/mixed lineage leukemia 1 (MLL1) chromatin modifying complexes, HOTTIP is involved in the enhancement of the transcription of multiple genes associated with the HOXA locus by promoting H3K27me3 histone modification [55,56]. In a study focusing on HOTTIP single-nucleotide polymorphisms (SNPs), the researchers found that HOTTIP rs1859168 A > C is significantly associated with a reduced PC risk, indicating the potential role of HOTTIP in PC [57]. In 2015, Li et al. described upregulated HOTTIP expression in PC tissues and cell lines and demonstrated that HOTTIP could promote proliferation, invasion, and GEM resistance both in vivo and in vitro. In addition, this research found that HOTTIP was positively correlated with HOXA13, and it was speculated that HOTTIP could play a cancer-promoting role in PC through HOXA13 [58]. Interestingly, Cheng et al. reached a different conclusion. They pointed out that HOTTIP in PC cells did not regulate HOXA13 but participated in regulating several other HOX genes, including HOXA10, HOXB2, HOXA11, HOXA9, and HOXA1 [59]. The cause of the inconsistency of HOTTIP-regulated genes between the two studies is still unknown, but the differences in the cell lines applied could be a possible reason. A recent study uncovered that the HOTTIP-mediated promotion of PC progression occurs in both HOXA13-dependent and HOXA13-independent manners and that HOTTIP expression is negatively regulated by miR-497 [60]. In addition to its role in cell proliferation, invasion, and chemoresistance, the function of HOTTIP in PC stem cells (CSCs) was also investigated. The authors analyzed the expression of HOTTIP in pancreatic CSCs and nonpancreatic CSCs in PC tissues by laser capture microdissection (LCM) and found that HOTTIP was highly expressed in pancreatic CSCs. Further functional assays showed that HOTTIP alterations affected pancreatic CSC stemness features, including tumorigenesis, sphericity, and stem factor (Lin28, Nanog, Oct4 and SRY-box transcription factor 2, Sox2) and marker (ALDH1, CD44 and CD133) expression. By binding to WDR5, HOTTIP promoted HOXA9 expression, which further activated the Wnt/β-catenin pathway [61].

LncRNA metastasis-associated lung adenocarcinoma transcript 1 (MALAT1) is also called nuclear-enriched abundant transcript 2 (NEAT2) and is located on chromosome 11q13; it is a highly evolutionarily conserved, 8.7-kb long non-coding transcript [62,63]. MALAT1 mainly acts as a molecular scaffold for various riboprotein complexes and functions as a transcriptional and epigenetic regulator [64,65,66]. To verify whether MALAT1 is dysregulated in PC, Liu et al. first detected MALAT1 expression in 45 PC tissues by qPCR. The results showed that MALAT1 was significantly higher in tumor tissues than in adjacent normal tissues. Higher MALAT1 expression was significantly correlated with an advanced tumor stage, a deeper invasion, and a shorter disease-free survival (DFS) time. Moreover, MALAT1 was found to be an independent predictor of DFS in PC patients [67]. These findings were further confirmed by subsequent studies [68,69]. Functional analysis clarified that MALAT1 is involved in promoting cell proliferation, migration, and invasion in vitro. After MALAT1 knockdown, G2/M cell cycle arrest and cell apoptosis were induced. Additionally, the suppression of epithelial-mesenchymal transition (EMT) and CSC-like properties were implicated [68]. In terms of the underlying mechanism, EZH2 was observed to be recruited to the E-cadherin promoter by MALAT1, where it prompted H3K27me3 at the E-cadherin promoter to repress its expression [70]. This mechanism is also responsible for the regulation of MALAT1-suppressed N-myc downregulated gene-1 (NDRG-1), which is a tumor suppressor in PC that is also coinhibited by EZH2 [71]. MALAT1 could also aid in increasing the proportion of pancreatic CSCs, maintaining the self-renewal abilities of cells, inducing chemoresistance, and promoting tumor angiogenesis. The internal mechanism may involve the self-renewal-related factor Sox2, but the detailed mechanism awaits further investigation [69]. In addition, the Hippo-YAP signaling pathway was also implicated in MALAT1-mediated PC progression [72].

### 4.2. LncRNAs Function as ceRNAs

**Figure 3:**
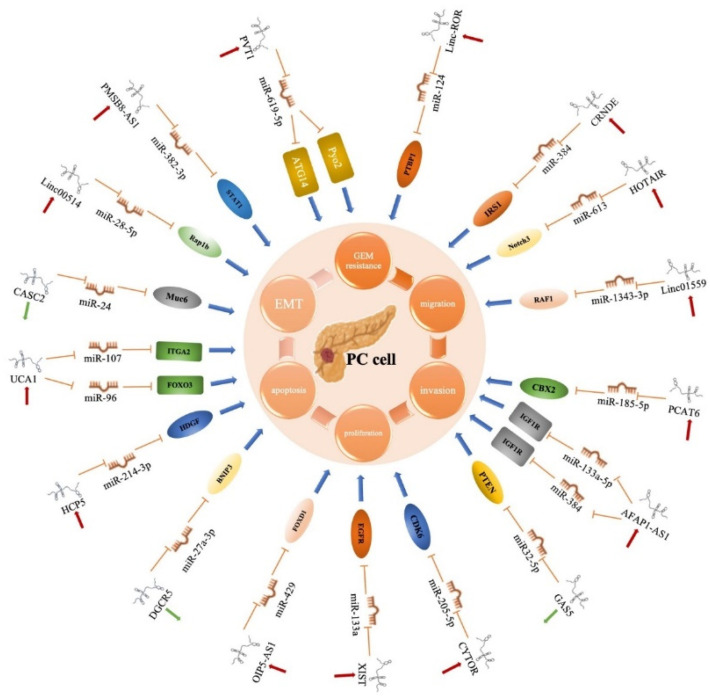
Overview of the role of lncRNAs with miRNAs in PC cells. By functioning as ceRNAs, lncRNAs can competitively bind to miRNAs, thereby blocking the inhibition of the target gene. PTBP1: polypyrimidine tract-binding protein 1; IRS1: insulin substrate receptor; CBX2: chromobox2; IGF1R: insulin-like growth factor 1 receptor; PTEN: phosphatase and tensin homolog deleted on chromosome ten; EGFR: epidermal growth factor receptor; FOXD1: forkhead box D1; BNIP3: Bcl-2/adenovirus E1B 19 kDa interacting protein 3; HDGF: hepatoma-derived growth factor; FOXO3: forkhead box O3; ITGA2: integrin subunit α 2; Muc6: mucin 6; STAT1: signal transducer and activator of transcription 1.

Various lncRNAs engaged in PC progression exert their functions through competing endogenous RNAs (ceRNAs), forming an interconnected lncRNA–miRNA–mRNA network. The following are several examples of lncRNAs acting as ceRNAs in PC (Figure 3).

Apart from its role in histone modification, MALAT1 also acts as a miRNA sponge. In liver fibrosis, MALAT1 regulates Rac1 expression by sponging miR-101b [73]. Luo et al. pointed out that TGFA-targeting miR-376A was sequestered by MALAT1 to promote osteosarcoma development [74]. It was found that miR-217 can bind MALAT1 and regulate its expression in PC cell lines. MALAT1 knockdown attenuated the protein expression of KRAS, a known target of miR-217. After MALAT1 deletion, the downregulation of KRAS expression could be attenuated by inhibiting miR-217. More importantly, MALAT1 knockdown did not directly affect cellular miR-217 expression but decreased the miR-217 nucleus/cytoplasm ratio, suggesting that MALAT1 inhibits the translocation of miR-217 from the nucleus to the cytoplasm [75]. Another study revealed that miR-200c-3p is also a target of MALAT1. By sponging miR-200c-3p, MALAT1 promoted the expression of the miR-200c-3p target zinc finger E-box-binding protein 1 (ZEB1), a known oncogenic factor. In turn, miR-200c-3p could suppress MALAT1 expression, thus uncovering a feedback loop between MALAT1 and miR-200c-3p expression [76]. In PC, researchers confirmed direct binding between miR-216a and MALAT1, and miR-216a was found to suppress MALAT1 expression. MiR-216a overexpression had a similar effect to MALAT1 siRNA in restoring p21 and p27 expression and inhibiting B-MYB, RAF1, and PCNA1 expression in PC cells. MiR-216a overexpression and MALAT1 knockdown induced cell cycle arrest at the G2/M phase. In addition, miR-216a also reduced cell viability and increased cell apoptosis in response to GEM in cancer cells. Based on these findings, we infer that miR-216a induces apoptosis in both the presence and absence of GEM in PC cells by silencing MALAT1 expression [77].

LncRNA H19 (H19) is located on human chromosome 11p15.5, and its role in cancer progression is still controversial. Considering the absence of H19 in embryonic rhabdomyosarcoma and Wilms’ tumor, H19 is thought to function as a tumor suppressor [78,79,80,81]. However, opposite findings were reported in other cancers, including bladder cancer, colon cancer, and gastric cancer, rendering H19 an oncogenic gene [82,83,84]. Ma et al. isolated pure malignant cells from frozen sections of PC tissues by LCM and detected that H19 was overexpressed in PC tissues and correlated with the histological grade of PC. The knockdown of H19 in T3M4 and PANC1 cells with high endogenous H19 levels suppressed cell viability, proliferation, and tumor growth. This led to G0/G1 arrest, accompanied by decreases in the levels of E2F transcription factor 1 (E2F1) and its downstream targets [85]. Additional studies revealed that H19 could facilitate PC cell invasion and migration by promoting high mobility group AT-hook 2 (HMGA2)-mediated EMT through antagonizing let-7 [86]. The H19/miR-194/CDK14 axis was also indicated to modulate PC proliferation and migration [87]. In addition to acting as an miRNA sponge, H19 could also influence PC by generating miRNAs embedded in its transcript. Researchers found that miR-675-5p, a miRNA transcribed simultaneously with H19, could contribute to PC progression by targeting E2F1 [88]. Interestingly, a recent study reported that, like miR-675-5p, miR-675-3p plays a role in H19-induced PC growth. By targeting STAT3, H19-derived miR-675-3p could activate SOCS5, thereby exerting an oncogenic effect [89].

LncRNA urothelial cancer-associated 1 (UCA1) is a noncoding RNA first identified to be associated with the tumorigenesis of bladder cancer [90]. Numerous studies have proven that UCA1 plays an oncogenic role in gastric cancer, breast cancer, and colorectal cancer [91,92,93,94]. One bioinformatic study focusing on PC in patients with diabetes pointed out that UCA1 was especially tied up with the prognosis of PC in patients with diabetes and could serve as a diagnostic marker [95]. By analyzing the lncRNA expression profiles in two public PC microarray datasets, researchers found that UCA1 might be involved in PC progression and was significantly associated with OS in PC [96]. Further functional experiments demonstrated that the downregulation of UCA1 could effectively suppress PC cell proliferative activities, increase the apoptotic rate and cause cell cycle arrest [97]. Regarding the mechanism, previous studies found that the overexpression of UCA1 could lead to the suppression of p27 protein and its downstream targets, thereby contributing to cell growth inhibition and apoptosis induction [92,98]. Subsequent studies reported that many miRNAs are tightly related to UCA1 in PC progression. A negative correlation was first identified between the expression of UCA1 and miR-107 by Gong et al., and then the luciferase reporter assay verified that miR-107 was targeted by UCA1. Further experiments confirmed that integrin subunit α 2 (ITGA2) was a target of miR-107. In downstream pathways, UCA1 and ITGA2 accelerate PC tumorigenesis via focal adhesion pathway-related proteins, including ITGA3 and protein tyrosine kinase 2 [99]. In addition, Zhou et al. observed the downregulation of miR-96 and further upregulation of its target FOXO3 by UCA1, and another UCA1-miR-590-3p-KRAS axis was identified by Liu et al. [100,101].

As a newly discovered lncRNA, lncRNA CYTOR (linc00152), was found to be aberrantly expressed in several malignant tumors [102,103]. Using next-generation sequencing and an extensive analysis of cDNA ends (MACE), the researchers found that CYTOR was differentially expressed between PC tissues and nontumor tissues [104]. Yu et al. analyzed the lncRNA expression profile in human PC tissues and nontumor tissues using four independent public microarray datasets from the Gene Expression Omnibus (GEO); this study confirmed that CYTOR presented a different expression pattern in PC tissues compared with normal tissues, which was further validated in PC cell lines and normal cells. Cell experiments showed that CYTOR could regulate PC cell proliferation and invasion [105]. This effect was later confirmed to be mediated by miR-205-5p, which directly targets CDK6 to control the proliferation and migration of PC cells [106]. A luciferase reporter assay also verified that miR-150 was a target of CYTOR. Moreover, the inhibition of miR-150 could significantly attenuate the suppression of cell proliferation, migration, and invasion induced by downregulating CYTOR [107]. Collectively, these findings suggest that CYTOR contributes to PC tumorigenesis by functioning as a sponge for multiple miRNAs.

### 4.3. LncRNAs Bind to Proteins

Several lncRNAs have been found to bind to specific proteins to exert their biological functions. By interacting with proteins, lncRNAs can modulate protein activity and stability and facilitate protein–protein interactions, thereby participating in cellular processes in PC [37].

As a novel lncRNA, lncRNA of metastasis suppressor 1 (MTSS1-AS) was downregulated in PC tissues and correlated with PC clinicopathology, including vascular infiltration, lymphatic invasion, and distant metastasis. Additionally, MTSS1-AS was inversely associated with OS in PC patients and could predict prognosis with an area under the curve (AUC) of 0.691 [108]. MTSS1-AS could scaffold the interaction between E3 ubiquitin-protein ligase STIP1 homology and U-box containing protein 1 (STUB1) and transcription regulator myeloid zinc finger 1 (MZF1), leading to MZF1 degradation by ubiquitination. Under acidic conditions, MTSS1-AS was downregulated, and this downregulation caused an increase in MZF1. MZF1 further suppressed MTSS1 expression, which was validated to be downregulated in PC and inversely correlated with disease progression [108]. Moreover, it was found that MTSS1-AS was transcriptionally repressed by binding the MYC proto-oncogene (Myc) with initiator (Inr) elements of the MTSS1-AS promoter. Reciprocally, MTSS1-AS blocked the expression of Myc by inhibiting MZF1-mediated Myc transcription, thereby forming a negative feedback loop between MTSS1-AS and Myc in acidic PC cells. This study provided us with a possible pathway responsible for the metastasis of PC cells under an acidic microenvironment [109].

Linc00673, reported in thyroid and tongue squamous cell carcinoma, is regarded as a tumor suppressor that inhibits the invasion and metastasis of tumor cells [110,111]. Utilizing a genome-wide association study (GWAS), researchers found that variation in linc00673 was correlated with PC risk. For example, a G > A change at rs11655237 in exon 4 of linc00673 creates a target site for miR-1231 binding, which reduces the effect of linc00673 in an allele-specific manner and thus confers susceptibility to tumorigenesis [112]. Further studies illustrated that linc00673 could suppress PC cell viability and migration by regulating hepatocyte nuclear factor 1 (HNF1A) via competitively binding to miR-504 [113]. Specifically, researchers found that linc00673 could promote the interaction between protein tyrosine phosphatase nonreceptor type 11 (PTPN11) and pre-mRNA-processing factor 19 (PRPF19), an E3 ubiquitin ligase, thereby promoting PTPN11 degradation through ubiquitination. PTPN11 degradation ultimately suppressed SRC-ERK oncogenic signaling and activated the signal transducer and activator of the transcription 1 (STAT1)-dependent antitumor pathway [112].

LncRNA nuclear-enriched abundant transcript 1 (NEAT1) is a lncRNA restricted to the nucleus. It functions as a transcriptional regulator of multiple genes, and its dysregulation is implicated in many human cancers [114,115,116,117,118,119]. Studies on PC showed that NEAT1 was highly expressed in PC and was associated with poor survival. Functional experiments revealed that NEAT1 remarkably suppressed cell proliferation and metastasis by regulating miR-506-3p and miR-302a-3p [120,121]. According to previous studies, E74-like ETS transcription factor 3 (ELF3) accelerates hepatocellular carcinoma progression by promoting EMT and metastasis [122]. In PC, NEAT1 was found to bind with ELF3 mRNA and enhance the interaction between insulin-like growth factor 2 mRNA-binding protein 1 (IGF2BP1) and ELF3 mRNA, thereby suppressing ELF3 mRNA degradation. Then, increased ELF3 was identified to promote PC cell migration and invasion [123].

A previous study reported that linc00346 was upregulated and served as a prognostic marker in PC [124]. Linc00346 could promote PC growth and GEM resistance by antagonizing miR-188-3p and inducing RD4 expression [125]. Another study using RNA precipitation assays together with mass spectrometry analysis revealed that linc00346 could promote the transcription of c-Myc via interaction with the CCCTC-binding factor (CTCF). This interaction between linc00346 and CTCF prevented CTCF from binding to the c-Myc promoter, blocking the CTCF-mediated inhibition of c-Myc. Thus, these results clarified that linc00346 contributes to PC pathogenesis by stimulating c-Myc expression [126].

### 4.4. LncRNAs Regulate the EMT Pathway in PC

EMT is the cellular process by which cells lose epithelial features and adopt a mesenchymal phenotype. This process endows the cells with properties of migration and metastasis. Accumulating studies have proven that EMT plays a critical role in the progression of malignant tumors and involves many transcription factors, including Snail, Twist-1, ZEB1/2, and Snai2. This process is accompanied by the increased expression of mesenchymal markers N-cadherin and vimentin and the decreased expression of epithelial markers E-cadherin [127,128]. Multiple lncRNAs have been verified to promote PC development via the EMT process in PC.

Studies have reported that lncRNA taurine-upregulated gene 1 (TUG1) mediates the progression of osteosarcoma and bladder cancer by stimulating cell proliferation [129,130]. TUG1 was expressed at higher levels in PC tissues than in paracarcinoma tissues and could enhance viability, migration, and GEM resistance-processes often facilitated by EZH2-in PC cells [131,132,133]. Significantly, the protein levels of matrix metalloproteinase 2 (MMP2) and MMP9 were increased, and the protein level of E-cadherin was decreased, indicating the enhancement of the EMT process. Mechanistically, overexpressed TUG1 could promote Smad2 and Smad3 phosphorylation. The expression of TGF-β and TGF-β receptors was higher in the TUG1 overexpression group than in the control group, indicating that the TGF-β/Smad pathway might participate in the TUG1-induced effect on PC cell EMT [134]. Another study found that TUG1 suppression could significantly increase miR-29c expression and thereby downregulate the expression of mesenchymal markers such as N-cadherin and vimentin but upregulate E-cadherin expression. The inhibition of miR-29c reversed tumor growth inhibition resulting from TUG1 knockdown both in vitro and in vivo, whereas miR-29c overexpression exhibited the opposite effects. Taken together, these findings indicate that TUG1 could modulate EMT by targeting the tumor suppressor miR-29c [135].

LncRNA, which is highly upregulated in liver cancer (HULC), is highly expressed in the tissues and serum of PC patients and is useful when distinguishing PC patients from patients with benign pancreatic diseases and healthy individuals [136]. By regulating the expression of proteins involved in the Wnt/β-catenin signaling pathway, including c-Myc, β-catenin, and CKD1, HULC is engaged in the proliferation, apoptosis, and invasion of PC cells [136]. Additionally, miR-15a-mediated activation of the PI3K/AKT signaling pathway was verified to be involved in the effects of HULC on PC cells [137]. Regarding the involvement of EMT, Takahashi et al. revealed that circulating extracellular vesicle (EV)-encapsulated HULC may act as a potent biomarker for detecting human PC and that lncRNA HULC could contribute to the invasion and migration of PC cells by inducing the EMT pathway [138]. In addition, HULC could be targeted by miR-133b in PC cells, which provides insight into the regulation of HULC biogenesis [138]. They further uncovered that during TGF-β induced EMT in PC cells, miR-622 was downregulated, which led to the HULC-mediated promotion of the invasion, migration and suppression of EMT. Moreover, miR-622-overexpressing EVs could transfer miR-622 to recipient PC cells, thus repressing the expression of HULC and inhibiting PC invasion and migration [139]. These results open up the possibility to develop treatment strategies targeting lncRNA-regulating miRNAs for PC.

The lncRNA plasmacytoma variant translocation 1 (PVT1) oncogene, located at chromosome 8q24.21, is upregulated in various cancers, including PC [140,141,142,143,144,145]. Interestingly, You et al., using a piggyBac transposon-based genome-wide mutagenesis strategy, identified that the PVT1 gene was associated with increased sensitivity to GEM in human PC cells [146]. The high expression of PVT1 could contribute to tumor progression in PC, and it could act as a potential biomarker to predict PC prognosis [141]. In PC, PVT1 upregulation significantly promoted ZEB1/Snail expression but inhibited p21 expression, and p21 downregulation further enhanced ZEB1/Snail expression and cell proliferation in PANC-1 cells. Therefore, PVT1 promoted EMT and cell proliferation and migration by downregulating p21 in PC cells [147]. Another study suggested that PVT1-regulated EMT was mediated through the TGF-β/Smad pathway [148]. Additionally, multiple miRNAs were implicated in PVT1 and PC, including miR-448, miR-519d-3p, and miR-20a-5p [149,150,151].

### 4.5. LncRNAs Modulate CSC Properties

CSCs are a subset of tumor cells with self-renew and differentiation abilities [152]. CSCs are engaged in tumor growth, metastasis, and drug resistance processes and are critical to the progression of many cancers [152,153,154,155,156]. The identification of CSCs is often dependent on the detection of stemness markers, mainly CD24, CD44, CD133, Nanog, Sox2, Sox9, OCT1/2/4, c-Myc, Kruppel-like factor 4 (KLF4), and essential specific antigen (ESA) [157,158,159]. LncRNAs have been illustrated to modulate CSC properties in diverse human cancers, including PC.

LincRNA-regulator of reprogramming (linc-RoR) is highly expressed in embryonic stem cells (ESCs) and induced pluripotent stem cells (iPSCs) and was initially confirmed to be a factor engaged in reprogramming differentiated cells to iPSCs, which is controlled by pluripotency transcription factors such as Sox2, Oct4, and Nanog [160,161,162]. In 2016, Zhan et al. first reported that linc-RoR was significantly upregulated in PC tissues and cell lines and contributed to cell proliferation, migration, invasion, and metastasis both in vitro and in vivo [163]. Mechanistically, they confirmed that linc-RoR could upregulate ZEB1, a factor shown to regulate EMT in many tumor cells, and this process is partially mediated by p53 [163,164,165,166]. Another study indicated that the activation of the Hippo/YAP pathway also played a role in linc-RoR-mediated EMT [167]. Additionally, linc-RoR could contribute to GEM resistance by inducing autophagy, a mechanism dependent on the targeting of PTB1 by miR-124 [168]. In PC, knockdown of linc-RoR in CSCs inhibited proliferation, induced apoptosis, decreased migration in vitro, and suppressed tumorigenicity in vivo. In this process, linc-RoR may act as a ceRNA to compete for miR-145 binding, thereby activating the derepression of the core transcription factor Nanog, which has previously been shown to play critical roles in maintaining stem cell pluripotency and iPSC reprogramming [169]. Fu et al. also observed that linc-RoR expression increased in cancer stem-like cells (CSLCs) and that linc-RoR knockdown impaired the properties and tumorigenesis of pancreatic CSLCs in vivo. They found that linc-RoR functioned as a ceRNA for several tumor suppressor miRNAs, particularly some members of the let-7 family [170].

Ultra-conserved RNAs (ucRNAs) are a group of lncRNAs with highly conserved sequences that are engaged in multiple biological functions [171,172,173,174]. In PC, a lncRNA termed ultraconserved element 345 (uc.345) was reported to be upregulated in tumor tissues, especially in tissues with an increased depth of invasion and advanced TNM stage, indicating that uc.345 could be an independent risk factor for the OS of PC patients. The authors employed soft agar assays and tumor xenograft models to show that uc345 could accelerate tumor growth. Regarding the mechanism, they found that the ratio of CD44+/CD24+ cells (which are recognized as CSCs) was upregulated by uc.345 overexpression. At the same time, pluripotency-related transcription factors such as Sox2, Oct4, Nanog, and CD133 were remarkably increased. These findings suggest that uc.345 promotes PC pathogenesis by increasing the proportion of CSCs [175]. Further exploration illustrated that this effect was attributed to the upregulated expression of hnRNPL, which is an RNA binding protein involved in tumorigenesis via multiple mRNA processing steps [176,177].

LncRNA AFAP1-AS1 is an antisense transcript of the actin filament-associated protein (AFAP1) gene, the sense strand of which encodes the AFAP1 protein. Using next-generation sequencing and MACE, researchers identified AFAP1-AS1 to be differentially expressed between PC tissues and control tissues [104]. The high expression of AFAP1-AS1 in PC tissues and cell lines was associated with lymph node and perineural invasion and poor survival, making it a promising prognostic factor for predicting tumor progression [178]. Functional experiments further suggested that the high expression of AFAP1-AS1 and activin receptor A type I (ACVR1) was accompanied by the low expression of miR-384, and AFAP1-AS1 silencing or miR-384 overexpression could impair PC cell self-renewal ability and stemness. Further research uncovered that the inhibitory effect of AFAP1-AS1 on PC cell stemness was exerted through binding to miR-384 to regulate ACVR1 expression [179]. Apart from miR-384, several other miRNAs were also involved in AFAP1-AS1-mediated PC progression, including miR-133a, miR-146b-5p, and miR-384. These findings provide additional valuable targets for investigating the role of AFAP1-AS1 in the CSC pathway in PC [96,179,180,181,182,183].

Human maternally expressed gene 3 (MEG3) is a lncRNA located at 14q32 with a nucleotide length greater than 1.6 kb [184]. An increasing number of studies have revealed that MEG3 functions as a tumor suppressor and the possible mechanism involves the modulation of cell growth and apoptosis [185,186,187,188]. In PC, MEG3 is downregulated and negatively correlated with tumor size, metastasis, and vascular invasion [189]. The involvement of the PI3K/AKT/Bcl-2/Bax/CKD1/P53 and PI3K/AKT/MMP2/MMP9 signaling pathways was implicated in the effect of MEG3 on PC progression [189]. Notably, the absence of MEG3 increased the sphere-forming ability and CSC properties of PC cells, whereas MEG3 overexpression led to the opposite effect. Snail activation was indicated as a component of the detailed mechanism [190].

The lncRNA Sox2 overlapping transcript (Sox2ot) gene is mapped to the human chromosome 3q26.3 locus and consists of highly conserved sequences of over 700 kb [191]. Containing a critic regulator of pluripotency, Sox2, in its intronic region, lncRNA Sox2ot has been explored in diverse somatic cancers, including esophageal squamous cell carcinoma, breast cancer, lung squamous cell carcinoma, and hepatocellular carcinoma [192,193,194,195]. The expression of Sox2ot in PC tissues and cell lines is strongly elevated, and functional experiments suggest that Sox2ot could act as a tumor promoter in PC by physically binding to FUS, thereby regulating its downstream proteins CCND1 and p27, which are regarded as cell cycle-associated factors [196]. More importantly, Li et al. identified that lncRNA Sox2ot could be derived from the exosomes of PC cells, and a positive association was revealed between Sox2ot expression in plasma exosomes and TNM stage and OS rate in PC patients. Further experiments discovered that by modulating Sox2 expression, Sox2ot promoted EMT and CSC-like properties in PC cells. Mechanistically, Sox2ot competitively binds to miR-200 family members, which further target Sox2 expression, thus enhancing the invasive and metastatic properties of PC. Moreover, the researchers found that tumor-derived exosomes could be transmitted to tumor cells or blood circulation in vivo, thereby translating the effect of Sox2ot from that of producer cells to that of recipient cells. In postoperative PC patients, decreased exosomal Sox2ot expression was also observed in blood samples, suggesting a promising role for exosomal Sox2ot as a marker for PC prognosis [197].

## 5. Clinical Significance of LncRNAs in PC

A large number of lncRNAs are aberrantly expressed in diverse cancers, and some have been verified to be cancer-specific. LncRNAs are usually detected in pancreatic tissues or body fluids such as plasma or saliva, and their expression is often related to disease severity. Thus, lncRNAs have the potential to serve as noninvasive biomarkers for cancer diagnosis and prognosis evaluation [198,199,200]. The following section lists several representative lncRNAs that could be applied as feasible diagnostic and prognostic biomarkers in PC (Table 4, Table 5 and Tables S3 and S4).

### 5.1. Diagnostic Biomarkers for PC

Xie et al. conducted the first study to investigate the clinical value of salivary lncRNA in the detection of PC [201]. Five well-documented lncRNAs, H19, HOTAIR, HOTTIP, MALAT1, and PVT1, which are most closely associated with PC from previous studies, were selected as putative lncRNA biomarkers. Compared with benign pancreatic tumor (BPT) and normal pancreatic tissues (NPT), HOTAIR, HOTTIP, and PVT1 were significantly upregulated in PC tissues. Compared to the BPT or healthy groups, the salivary levels of HOTAIR and PVT1 were substantially higher in the PC group. After curative pancreatectomy, the salivary levels of HOTAIR and PVT1 were reduced considerably. ROC analysis showed that both salivary lncRNAs could distinguish PC patients from healthy controls and BPT patients with sensitivities and specificities ranging from 60–97%. The expression of salivary HOTAIR and PVT1 did not differ significantly between healthy controls in any one of eight leading cancers worldwide. In addition, the two lncRNAs in saliva showed better discriminatory power in detecting PC with serum CA19-9 < 37 U/mL from healthy controls. Collectively, these findings indicate that salivary HOTAIR and PVT1 have the potential to become novel noninvasive biomarkers for detecting PC [201]. A more recent study explored the diagnostic value of plasma HOTAIR in PC and reported an AUC of 0.9329, suggesting that plasma HOTAIR could serve as an ideal biomarker for detecting PC [54]. Studies on MALAT1 illustrated that MALAT1 expressed in the tissue could distinguish PC tissues from normal tissues with an AUC of 0.69 (95% CI 0.561~0.829, *p* = 0.009), and the sensitivity and specificity values reached 77.8% and 60%, respectively [67]. Moreover, data from the Oncomine, the GEO, and The Cancer Genome Atlas databases revealed a moderate diagnostic value of MALAT1 in PC (AUC = 0.75, sensitivity = 0.66, specificity = 0.72) [202].

GWAS showed that rs6971499 at 7q32.3 (linc-pint) was significantly differentially expressed at the genome level between PC patients and normal controls [203]. Researchers used qRT-PCR and RNA FISH analysis to evaluate linc-pint levels in the plasma and tumor tissues of PC patients and found that linc-pint expression was lower in the plasma samples from PC patients than in those of healthy individuals, and plasma linc-pint levels could detect PC with higher sensitivity than CA19-9. The data also showed that plasma linc-pint levels were lower in PC patients than in a patient with carcinoma of the ampulla of Vater (CAV) and cholangiocarcinoma (CCA) and therefore could be used to distinguish the cause of malignant obstructive jaundice. In addition, low plasma linc-pint levels were correlated with tumor recurrence. The levels of linc-pint were lower in PC tissues than in adjacent tissues and CAV and CCA tissues and were associated with a poor prognosis for PC patients after pancreatectomy [204]. Similar results were also reported in studies conducted by Lu et al., supporting linc-pint as a diagnostic parameter in PC [205].

LncRNA CCDC26 (CCDC26), located on chromosome 8q24, has been reported to have a tumorigenic role of in many malignant tumors [206,207,208]. CCDC26 is highly expressed in PC tissues compared with normal tissues, and its expression is correlated with tumor size, tumor number, and reduced OS. Additionally, CCDC26 expression was identified as an independent prognostic factor in terms of OS in PC patients. Importantly, ROC analysis showed that the AUC of CCDC26 was as high as 0.663, indicating that CCDC26 could be a diagnostic marker for distinguishing PC tissues from healthy tissues.

### 5.2. Prognostic Biomarkers for PC

LncRNA opa-interacting protein 5 antisense RNA 1 (OIP5-AS1) has been demonstrated to play an oncogenic role in the tumorigenesis of many cancer types [209,210,211,212]. Wu et al. used 110 pairs of PC tissues and adjacent normal tissues collected from PC patients after surgery and revealed that OIP5-AS1 is upregulated in tumor tissues and is positively correlated with tumor size, distant metastasis, and TNM stage. Kaplan–Meier survival analysis showed that patients with a high expression of OIP5-AS1 had poorer OS. Moreover, multivariate Cox regression analysis validated OIP5-AS1 expression and TNM stage as independent prognostic factors for PC patients [213]. Another study revealed higher OIP5-AS1 expression in metastatic and advanced-stage tumors [214].

According to previous studies, lncRNA X-inactive specific transcript (XIST) is involved in the development and progression of many malignant tumors [215,216,217,218]. Numerous studies have reported that lncRNA-XIST is upregulated in PC tissues and cell lines, and high XIST expression in PC is related to poorer prognosis (larger tumor size, perineural invasion, lymph node micrometastases, and shorter OS) [219].

LncRNA cancer susceptibility candidate 2 (CASC2) has been widely defined as a tumor suppressor in various cancers [220,221,222,223,224,225]. Yu et al. found that lncRNA-CASC2 was specifically downregulated in PC tissues and cell lines, and lower CASC2 expression in PC was related to a poorer prognosis [226]. This relationship was later confirmed by subsequent studies [227,228]. Linc00671 is an 1844-bp lncRNA and is located on chromosome 17q21.31. Compared with normal tissues, PC tissues had decreased linc00671 expression. Correlation analysis revealed a negative association between linc00671 expression and tumor differentiation, clinical stage, and a poor prognosis [229]. ROC curve analysis showed that the AUC of linc00671 in detecting PC was 0.6057, and high linc00671 expression was related to a significantly higher survival rate than low expression [124].

Due to the late diagnosis and low resection rate of PC, chemotherapy remains a critical strategy in PC treatment. Among various chemotherapy agents, GEM is the first-line therapeutic choice approved for advanced PC treatment, and it can be used both alone and in combination with other chemotherapeutic drugs [230]. Because of acquired and/or inherent resistance, the efficacy of GEM in improving the OS rates of PC is not gratifying [231,232]. Therefore, it is imperative to understand the internal mechanism of GEM resistance to achieve better benefits from cancer therapy [178]. Numerous reports have shown that lncRNAs could contribute to GEM resistance in PC.

Previous studies have revealed that PVT1 is involved in the processes of carcinogenesis and chemoresistance [233]. Further studies revealed that knockdown of PVT1 could sensitize cancer cells to GEM, while the overexpression of PVT1 blocked this effect in PC cells [146]. Regarding the detailed mechanism, researchers found that GEM could increase the expression of drosha ribonuclease III (drosha) and DGCR8 microprocessor complex subunit (DGCR8) to promote the generation of the miR-1207 pair from the PVT1 transcript, thereby disrupting oncogenic signaling in PC cells by targeting ras homolog family member A (RhoA) and the SRC proto-oncogene (nonreceptor tyrosine kinase) [234]. More recently, a study elaborated that PVT1-mediated drug resistance could be attributed to the activation of Wnt/β-catenin signaling and autophagic activity [235]. They found that by decoying miR-619-5p, PVT1 could upregulate the expression of Pygo2 and ATG14, which are responsible for activating both Wnt/β-catenin signaling and the autophagic pathway. In return, elevated Wnt/β-catenin signaling activates PVT1 expression by directly binding to the PVT1 promoter, thereby forming a positive feedback loop between PVT1 expression and Wnt/β-catenin signaling [235]. Moreover, through the interaction with ATG14, PVT1 facilitates the assembly of autophagy-specific complex I (PtdIns3K-C1) and the activation of ATG14-dependent class III PtdIns3K, thereby mediating GEM resistance in PC [235].

LncRNA human histocompatibility leukocyte antigen complex P5 (HCP5), initially identified to be expressed in immune system cells, has been explored in diverse human cancers [236,237,238,239,240]. In PC, by utilizing the GSE15471 and GSE16515 datasets and the Database for Annotation, Visualization, and Integrated Discovery (DAVID), Wang et al. conducted a functional enrichment analysis which demonstrated that the MMP9/ITGB1-miR-29b-3p-lncRNA HCP5 network was associated with the prognosis of PC [241]. A recent study found that lncRNA HCP5 was highly expressed in PC tissues and prompted PC cell proliferation, migration, and invasion by targeting miR-140-5p and upregulating CDK8 expression [242]. Moreover, lncRNA HCP5 was prominently increased in GEM-resistant PC tissues and cells, indicating the critical role of HCP5 in GEM resistance. Cytological experiments revealed that suppressed lncRNA HCP5 could affect the proliferation, invasion, apoptosis, and autophagy properties of GEM-resistant PC cells. This was further confirmed to be fulfilled by inhibiting HDGF expression through sponging miR-214-3p. Therefore, lncRNA HCP5 may represent a potential treatment target for GEM-resistant PC [243].

LncRNA growth arrest-specific 5 (GAS5) is a newly discovered lncRNA that facilitates cell proliferation, the cell cycle, and chemoresistance [244,245,246]. It has been reported that GAS5 is downregulated in PC tissues and cell lines [247]. In vitro experiments illustrated that GAS5 modulates the expression of CDK6, which is responsible for the regulation of cell proliferation and the cell cycle [248]. Another study showed that upon inhibiting GAS5, CD133+ cells, responsible for tumor recurrence, were released from growth arrest and exhibited accelerated nucleic acid biosynthesis and proliferation [249]. Regarding chemoresistance, researchers have reported that GAS5 negatively regulates miR-181c-5p and that miR-181c-5p can remarkably enhance PC cell chemoresistance by inhibiting the Hippo signaling pathway [250]. Another study conducted by Liu et al. clarified that GAS5 could directly bind to miR-221 and downregulate miR-221 expression in GEM-resistant PC cells. Gemcitabine resistance induced by miR-221 overexpression could be successfully attenuated by SOCS3 overexpression [251]. Taken together, these findings indicate that lncRNA GAS5 functions as a ceRNA for miR-181c-5p and miR-221 to suppress the development of chemoresistance in PC progression.

In addition to gemcitabine, the role of lncRNA in other forms of drug resistance during PC treatment has also been reported. A recent study uncovered that lncRNA UPK1A-AS1 expression was associated with a poor oxaliplatin-based chemotherapeutic response and a shorter PFS time in advanced PC patients. Mechanistically, IL8 secreted by cancer-associated fibroblasts (CAFs) could induce the expression of UPK1A-AS1, which further strengthened the interaction between Ku70 and Ku80 to facilitate nonhomologous end-joining (NHEJ), thereby enhancing DNA double-strand break (DSB) repair. Therefore, UPK1A-AS1 mediated CAF-derived paracrine IL8-dependent oxaliplatin resistance implicates a potential therapeutic target [252].

## 6. Future Expectations

Considering that lncRNAs play indispensable roles in tumor pathogenesis, it is of great significance to design potential diagnostic and therapeutic strategies targeting lncRNAs to gain control of malignant tumors. One feasible method is to interfere with lncRNA expression with siRNAs or DNA plasmids, which is the most widely used method in basic research to regulate lncRNAs [35,253]. In 2009, Mizrahi A et al. designed a DNA plasmid called H19-DTA, which contains the diphtheria toxin-A gene to target H19 expression. In vivo experiments showed that H19-DTA could suppress the growth of multiple cancer types [254]. Later, two clinical trials were conducted to verify the efficacy of H19-DTA in cancer patients, where both studies observed suppressed tumor growth and prolonged survival times [255,256]. However, to date, no clinical trials of H19-DTA focusing on PC have explicitly been documented, so whether H19-DTA could exert similar effects is PC still awaits further exploration.

Recently, the development of clustered regulatory interspaced short palindromic repeats/CRISPR-associated protein 9 (CRISPR-Cas9) technology and its use in a variety of diseases have drawn much attention [257,258]. Despite the limitations in this field, ncRNA editing using CRISPR-Cas9 technology has been explored in various cancer types [259]. Zhen et al. found that silencing lncRNA UCA1 via the CRISPR/Cas9 method could effectively block bladder cancer progression [260]. It is worth noting that lncRNA UCA1 is also highly expressed in PC, and the downregulation of UCA1 could effectively suppress PC cell proliferation, promote apoptosis, and induce cell cycle arrest [97]. These clues prompted us to reflect on the possibility of inhibiting UCA1 expression with CRISPR/Cas9 technology to treat PC. To date, no investigation on CRISPR-Cas9 editing lncRNA in PC has been reported, but it is foreseeable that this approach could become a promising strategy for PC treatment in the near future.

Strategies for safely, efficiently, and continuously transporting stably altered lncRNAs to target cells or organs are also needed for the future application of lncRNAs. In recent years, exogenous nanoparticles, which can act as carriers for novel genes and drugs, have attracted wide attention [261]. Compared with traditional treatments, nanoparticles can reduce the concentration of a drug needed for effects that are otherwise only achieved with a high drug or radiation dose while increasing their distribution in target organs and avoiding systemic damage [262]. Another emerging approach for targeting lncRNAs is exosomes, which are defined as microvesicles with diameters of 30-100 nm that can be released from cells to exert intercellular communication functions [263]. Many studies have shown that exosomes containing lncRNAs can regulate the proliferation, migration, and invasion of PC cells [197]. Despite the tremendous progress made in these areas, these findings are still theoretical, and no treatment based on nanoparticles or exosomes has yet been approved in the clinic.

The early diagnosis of PC is crucial for improving the 5-year survival rate [264]. A large number of studies have shown that lncRNAs can serve as ideal noninvasive biomarkers for the diagnosis and prognosis of PC. In the future, a gold standard lncRNA detection method should first be identified to standardize the detection of lncRNAs in different laboratories. Additionally, multicenter, multipopulation trials with large sample sizes should be carried out to obtain more clinically significant thresholds. In practice, it should also be noted that PC development is a long-term and chronic process. The role of lncRNAs in the early diagnosis of precancerous lesions, including pancreatic intraepithelial neoplasia (PanIN), intraductal papillary mucinous neoplasm (IPMN), and mucinous cystadenoma (MCN), should be emphasized. Finally, noninvasive or minimally invasive detection methods are the ultimate goal. Trials related to lncRNA detection in peripheral blood and endoscopic biopsy samples (pancreatic juice or tissue) should be carried out in the early stages of clinical research.

## 7. Conclusions

Despite extensive efforts made in recent years to treat PC based on surgery, radiation, and chemotherapy, PC remains the seventh most deadly cancer worldwide; thus, the clinical practice of PC requires better biomarkers and treatment strategies. With the help of new detection technologies, studies focusing on lncRNAs have become a hotspot in the field of biological science, especially in the study of diverse cancers. This review summarizes the biogenesis, classification, and mode of action of lncRNAs, as well as the functions and mechanisms of lncRNAs in PC. Additionally, the clinical significance of ncRNAs in PC was discussed. However, what we have uncovered is only the tip of the iceberg, and there are still some obstacles on the way to a deeper understanding of the role of lncRNAs in PC. For example, recent studies have mainly focused on the ceRNA functions of lncRNAs. In contrast, the interactions between lncRNAs and other molecules, especially between lncRNAs themselves, have been rarely reported. Furthermore, to date, no lncRNA has been approved for the diagnosis or treatment of PC. Therefore, there is still a long way to go before these findings can be translated from the bench to the bedside. Nevertheless, with the continued emergence of more gratifying investigations, we believe that this will happen in the near future.

## Figures and Tables

**Table 1:** Classifications of lncRNAs.

Criterial	Classification
genome location	intergenic lncRNAs
	intronic lncRNAs
	exonic lncRNAs
transcriptional orientation	sense lncRNAs
	antisense lncRNAs
subcellular localization	nuclear lncRNAs
	cytoplasmic lncRNAs
mode of action	cis-acting lncRNAs
	trans-acting lncRNAs

**Table 2:** Overview of cellular functions of tumor-suppressive lncRNAs in pancreatic cancer.

No	Lnc	Vitro Functions									Specimen	Expression	Reference PMID
		Prolife ^a^	Cycle ^b^	Apopt ^c^	Migra ^d^	Invas ^e^	Angio ^f^	CSC ^g^	Drug Resistance	Other			
1	GAS5	+	+								BXPC3/PANC1/ASPC1/HS766T	down	24026436
2	ENST0000048073					+					ASPC1/BXPC3/CFPAC1/PANC1/SW1990	down	25314054
3	linc00673	+	+								BXPC3/CFPAC1	down	27213290
4	ENSG00000218510					+					ASPC1/BXPC3/CAPAN2/CFPAC1/HPAC/MIA PACA2/PANC1/SW1990	down	27628540
5	F11AS1										SW1990/BXPC3	down	28220683
	linc00226												
6	MEG3	+	+	+	+	+					PANC1	down	28320094
7	CASC2	+									SW1990/BXPC3/ASPC1/CFPAC1/PANC1	down	28865121
8	DAPK1				+	+					PANC1/HS766T	down	31966799
9	GAS5	+							GEM/5-FU ^h^		PATU8988/SW1990/PATU89885-FU/SW1990-GEM	down	29112934
10	DGCR5	+			+	+			5-FU		HPAC/PANC1	down	29207609
11	GAS5	+		+	+	+					BXPC3/PANC1	down	29225772
12	MEG3	+			+	+		+	GEM		MIAPACA2/PANC1/T3M4	down	29328401
13	XLOC_000647	+				+					BXPC3/MIAPACA2	down	29386037
14	AB209630	+							GEM		BXPC3-GEM/PANC1-GEM	down	29526843
15	BC032020	+	+	+	+						ASPC1/PANC1	down	29532883
16	PCTST	+				+					BXPC3/MIAPACA2	down	29978472
17	KCNK15AS1				+	+					BXPC3/MIAPACA2	down	30032148
18	linc00671/00261/SNHG9	+									BXPC3/HPAC	down	30210701
19	NONHSAT105177	+			+					cholesterol pathway/melittin/exosome	PATU8988/SW1990	down	30237397
20	GAS5	+		+	+			+	GEM		PANC1	down	30388621
21	GLSAS	+			+	+				glycolysis/glutamate	BXPC3/PANC1	down	30563888
22	CASC2				+	+					PANC1	down	30675129
23	linc00052	+	+	+	+	+					ASPC1/SW1990	down	30712321
24	TUSC7	+		+	+	+		+			CAPAN2/PANC1	down	30714151
25	linc-pint	+									HPNE/PL45	down	30944652
26	linc01197	+									ASPC1/BXPC3/HPNE/PANC1	down	31027497
27	PXNAS1	+				+					ASPC1/PACA2	down	31488171
28	GAS5	+				+		+	GEM	oxidation reaction	CD133HI(SU86.86/KPC001/S2VP10)/CD133LO(MIAPACA2)	down	31740660
29	linc01111	+	+		+	+					PANC1/MIAPACA2	down	31767833
30	CASC2	+		+	+	+					ASPC1/PANC1	down	31894271
31	linc00673	+		+	+						CAPAN1	down	31949497
32	linc00261				+	+					PANC1/MIAPACA2	down	32020223
33	linc285194			+	+					involved in the inhibition of PANC1 by propofol	PANC1	down	32303144
34	linc00261				+	+					A549/PANC1	down	32414223
35	linc00261	+		+		+					CFPAC1/BXPC3/PANC1/ASPC1	down	32590069
36	DGCR5			+							SW1990/PANC1	down	32626951
37	linc00671	+			+	+					PANC1/CAPAN2/CAPAN1/ASPC1/SW1990/BXPC3	down	32801328
38	MTSS1-AS				+	+					ASPC1/PANC1/SW1990/BXPC3	down	32929338

^a^. Prolife: proliferation; ^b^. Cycle: cell cycle; ^c^. Apopt: apoptosis; ^d^. Migra: migration; ^e^. Invas: invasion; ^f^. Angio: angiogenesis; ^g^. CSC: cancer stem cell; ^h^. GEM: gemcitabine. “+” means that the lncRNA plays a role in related cancer cell biological functions.

**Table 3:** Overview of mechanisms and animal studies of tumor-suppressive lncRNAs in pancreatic cancer.

No	Lnc	Position	Location	Mechanism					Animal Model	Vivo Functions		Pheno ^f^	Reference PMID
				miRNA ^c^	Target	RBPs ^d^	Pathways	Upstream		Grow	Meta ^e^		
1	GAS5				CDK6							suppr ^g^	24026436
2	ENST0000048073						OS-9/HIF-1a					suppr	25314054
3	linc00673	17q24.3				PTPN11	SRC/ERK; STAT1					suppr	27213290
4	ENSG00000218510											suppr	27628540
5	F11-AS1											suppr	28220683
	linc00226												
6	MEG3					PI3K	PI3K/AKT					suppr	28320094
7	CASC2						PTEN/AKT	HNF1A				suppr	28865121
8	DAPK1			miR-812			ROCK-1/RhoA					suppr	31966799
9	GAS5			miR-181c-5p	Hippo				subcutaneous xenograft	+		suppr	29112934
10	DGCR5			miR-320a			EMT					suppr	29207609
11	GAS5			miR-32-5p	PTEN				subcutaneous xenograft	+		suppr	29225772
12	MEG3	14q32.3					EMT; PCNA					suppr	29328401
13	XLOC_000647					NLRP3	EMT		subcutaneous xenograft	+		suppr	29386037
14	AB209630						PI3K/AKT					suppr	29526843
15	BC032020						ZENF451/TGF-β		subcutaneous xenograft	+		suppr	29532883
16	PCTST					TACC-3	EMT		subcutaneous xenograft	+		suppr	29978472
17	KCNK15-AS1		Nucl ^a^				EMT	ALKBH5				suppr	30032148
18	linc00671/00261/SNHG9											suppr	30210701
19	NONHSAT105177		nucl				EMT		subcutaneous xenograft	+		suppr	30237397
20	GAS5			miR-221	SOCS3	EMT			subcutaneous xenograft	+	+	suppr	30388621
21	GLS-AS	2	nucl			GLS premRNA	ADAR1/Dicer	Myc	subcutaneous xenograft	+	+	suppr	30563888
22	CASC2	10q26		miR-21	PTEN							suppr	30675129
23	linc00052			miR-330-3p					subcutaneous xenograft	+		suppr	30712321
24	TUSC7	3q13.31		miR-371a-5p			EMT		subcutaneous xenograft	+		suppr	30714151
25	linc-pint						TGF-β1					suppr	30944652
26	linc01197		nucl			β-catenin	TCF4; Wnt pathway	FOXO1	subcutaneous xenograft	+		suppr	31027497
27	PXN-AS1			miR-3046	PIP4K2B				subcutaneous xenograft	+		suppr	31488171
28	GAS5					GR	CDK4/CCND2; NF-kB	SOX2	KPC mice	+		suppr	31740660
29	linc01111		Cyto ^b^	miR-3924	DUSP1		SAPK/JNK		subcutaneous xenograft	+	+	suppr	31767833
30	CASC2			miR-24	MUC6		ITGB4/FAK; EMT		subcutaneous xenograft	+		suppr	31894271
31	linc00673			miR-504	HNF1A				subcutaneous xenograft	+		suppr	31949497
32	linc00261			miR-552-5p	FOXO3		Wnt/EMT		liver metastasis model		+	suppr	32020223
33	linc285194			miR-34a			E-cadherin					suppr	32303144
34	linc00261		nucl			E-cadherin		FOXA2				suppr	32414223
35	linc00261			miR-23a-3p								suppr	32590069
36	DGCR5			miR-27a-3p	BNIP3		p38 MAPK		subcutaneous xenograft	+		suppr	32626951
37	linc00671	17q21.31	cyto				EMT		subcutaneous xenograft	+		suppr	32801328
38	MTSS1-AS		nucl			MZF1						suppr	32929338

^a^. nucl: nucleus; ^b^. cyto: cytoplasm; ^c^. miRNAs: microRNA; ^d^. RBPs: RNA binding proteins; ^e^. Meta: metastasis; ^f^. Pheno: phenomenon; ^g^. suppr: suppressor. “+” means that the lncRNA plays a role in related cancer cell biological functions.

**Table 4:** Overview of clinicopathological significance of tumor-suppressive lncRNAs in pancreatic cancer.

No	Lnc	Source	No. of Patients	Express	Cut-Off	Variables								Reference PMID
						Size	Differentiation	T	Lymphatic (N)	Distance (M)	TNM	Vessel Invasion	Other	
1	GAS5	tissue	23	down										24026436
2	BC008363	tissue	30	down										25200694
3	ENST00000480739	tissue	35	down					+		+			25314054
4	LOC285194	tissue	85	down					+	+	+			25550852
5	linc00673	tissue	74	down										27213290
6	HMlincRwNA717	tissue	150	down		+			+	+	+			27338046
7	ENSG00000218510	tissue	80	down		+	+			+				27628540
8	linc-pint	plasma	59	down										27708234
		tissue	61	down										
9	MEG3	tissue	30	down		+				+		+		28320094
10	CASC2	tissue	110	down				+						28865121
11	DGCR5	tissue	30	down	MEL ^a^									29207609
12	GAS5	tissue	22	down										29225772
13	MEG3	tissue	25	down										29328401
14	XLOC_000647	tissue	48	down	MEL				+		+			29386037
15	AB209630	tissue	53	down										29526843
16	BC032020	tissue	20	down										29532883
17	PCTST	tissue	48	down	MEL						+			29978472
18	KCNK15-AS1	tissue	69	down										30032148
19	linc00261	tissue	229	down					+		+			30210701
20	linc00671	tissue	229	down					+		+			30210701
21	linc00671/00261/SNHG9	plasma	229	down										30210701
22	SNHG9	tissue	229	down					+	+	+			30210701
23	NONHSAT105177	tissue	N/A ^b^	down										30237397
24	GAS5	tissue	60	down										30388621
25	GLS-AS	tissue	30	down	MEL	+			+	+				30563888
26	TUSC7	tissue	94	down			+		+	+	+		perineural invasion	30714151
27	linc-pint	plasma	46	down		+								30944652
28	linc01197	tissue	18	down										31027497
29	PXN-AS1	tissue	50	down		+			+		+			31488171
30	linc01111	tissue	60	down					+		+			31767833
31	linc01111	plasma	57	down										31767833
32	CASC2	tissue	20	down										31894271
33	linc00673	tissue	30	down	MEL					+				31949497
34	DAPK1	tissue	60	down			+		+		+			31966799
35	linc00261	tissue	54	down	MEL	+			+	+	+	+	perineural invasion	32020223
36	linc00261	tissue	42	down	17.66		+				+			32414223
37	linc00671	tissue	60	down			+				+			32801328
38	MTSS1-AS	tissue	132	down					+	+		+		32929338

^a^. MEL: median expression level; ^b^. N/A: not available. “+” means that the lncRNA plays a role in related PC clinicopathological features.

**Table 5:** Overview of prognostic and diagnostic significance of tumor-suppressive lncRNAs in pancreatic cancer.

No	Lnc	Source	No. of Patients	Expression	Detection Method	AUC ^a^	Survival	Prognostic Biomarker	Reference PMID
1	GAS5	tissue	23	down	qRT-PCR				24026436
2	BC008363	tissue	30	down	qRT-PCR		OS ^b^		25200694
3	ENST00000480739	tissue	35	down	qRT-PCR		OS	+	25314054
4	LOC285194	tissue	85	down	qRT-PCR		OS	+	25550852
5	linc00673	tissue	74	down	qRT-PCR				27213290
6	HMlincRNA717	tissue	150	down	qRT-PCR		OS	+	27338046
7	ENSG00000218510	tissue	80	down	qRT-PCR	0.931	OS	+	27628540
8	linc-pint	plasma	59	down	qRT-PCR	0.87			27708234
		tissue	61	down	FISH and qRT-PCR		OS	+	
9	MEG3	tissue	30	down	qRT-PCR				28320094
10	CASC2	tissue	110	down	qRT-PCR		OS/DFS ^c^	+	28865121
11	DGCR5	tissue	30	down	qRT-PCR	0.735	OS		29207609
12	GAS5	tissue	22	down	qRT-PCR				29225772
13	MEG3	tissue	25	down	qRT-PCR		OS		29328401
14	XLOC_000647	tissue	48	down	qRT-PCR		OS	+	29386037
15	AB209630	tissue	53	down	qRT-PCR		OS		29526843
16	BC032020	tissue	20	down	qRT-PCR				29532883
17	PCTST	tissue	48	down	qRT-PCR		OS	+	29978472
18	KCNK15-AS1	tissue	69	down	qRT-PCR				30032148
19	00261	tissue	229	down	qRT-PCR	0.5712	OS		30210701
20	00671	tissue	229	down	qRT-PCR	0.6057	OS		30210701
21	00671/00261/SNHG9	plasma	229	down	qRT-PCR				30210701
22	SNHG9	tissue	229	down	qRT-PCR	0.5983	OS		30210701
23	NONHSAT105177	tissue	N/A ^d^	down	RNA-FISH				30237397
24	GAS5	tissue	60	down	IHC and qRT-PCR				30388621
25	GLS-AS	tissue	30	down	northern blot and qRT-PCR		OS		30563888
26	TUSC7	tissue	94	down	qRT-PCR		OS	+	30714151
27	pint	plasma	46	down	qRT-PCR	0.8934			30944652
28	01197	tissue	18	down	qRT-PCR		OS/DFS		31027497
29	PXN-AS1	tissue	50	down	qRT-PCR		OS		31488171
30	01111	tissue	60	down	ISH and qRT-PCR		OS		31767833
31	01111	plasma	57	down	qRT-PCR				31767833
32	CASC2	tissue	20	down	qRT-PCR				31894271
33	00673	tissue	30	down	qRT-PCR		OS		31949497
34	DAPK1	tissue	60	down	qRT-PCR				31966799
35	00261	tissue	54	down	qRT-PCR		OS		32020223
36	00261	tissue	42	down	qRT-PCR		OS		32414223
37	linc00671	tissue	60	down	qRT-PCR		OS		32801328
38	MTSS1-AS	tissue	132	down	qRT-PCR		OS		32929338

^a^. AUC: area under the curve; ^b^. OS: overall survival; ^c^. DFS: disease-free survival; ^d^. N/A: not available. “+” means that the lncRNA could be used as a prognostic biomarker in PC.

## Data Availability

Not applicable.

## Supplementary Materials

The following supporting information can be downloaded at: https://www.mdpi.com/article/10.3390/cancers14092115/s1, Table S1: Overview of cellular functions of oncogenic lncRNAs in pancreatic cancer; Table S2: Overview of mechanisms and animal studies of oncogenic lncRNAs in pancreatic cancer; Table S3: Overview of clinicopathological significance of oncogenic lncRNAs in pancreatic cancer; Table S4: Overview of prognostic and diagnostic significance of oncogenic lncRNAs in pancreatic cancer.
