# Supplementary material for: Long Non-Coding RNAs in Pancreatic Cancer: Biologic Functions, Mechanisms, and Clinical Significance
Source: Cancers (Basel). 2022 Apr 24;14(9):2115. doi: 10.3390/cancers14092115 (PMC9100048; doi:10.3390/cancers14092115)
Supplement: Supplementary file 1 [file cancers-14-02115-s001.zip › Supplementary tables.pdf]

Table S1. Overview of cellular functions of oncogenic lncRNAs in pancreatic cancer.

No	Lnc	Vitro Functions									Specimen	Expression	Reference PMID
		Prolife[a]	Cycle[b]	Apopt[c]	Migra[d]	Invas[e]	Angio[f]	CSC[g]	Drug resistance	Other			
1	HOTAIR	+	+	+		+					PANC1/MIAPACA2/PANC28/L3.6PL	up	22614017
2	MALAT1	+	+	+	+	+		+			BXPC3/CFPAC1/CAPAN1/SW1990/ASPC1/PANC1/HS766T	up	25269958
3	MALAT1			+				+	GEM[h]		ASPC1/CFPAC1	up	25811929
4	HOTTIP	+	+			+			GEM		PANC1/MIAPACA2/CAPAN2/SW1990/BXPC3	up	25889214
5	HOTTIP	+		+	+						PANC28/L3.6PL/PANC1/ASPC1/BXPC3/MIAPACA2	up	25912306
6	AFAP1-AS1	+			+	+					PANC1/MIAPACA2/CAPAN2/SW1990/BXPC3	up	25925763
7	AF339813	+	+	+							SW1990/PANC1/BXPC3	up	26045769
8	linc00675	+	+			+					PANC1/CAPAN2/BXPC3/MIAPACA2/SW1990	up	26309360
9	MIR31HG	+	+	+		+					ASPC1/PANC1/CFPAC1/HS766T/SW1990	up	26549028
10	linc-ROR	+	+	+	+			+			PANC1/CAPAN1/MIAPACA2/BXPC3/SW1990	up	26636540
11	LOC389641	+		+	+	+					SW1990/ASPC1/PANC1/MIAPACA2/BXPC3/CAPAN2	up	26708505
12	NUTF2P3-001	+				+					PANC1/BXPC3	up	26755660
13	linc-ROR	+			+	+					ASPC1/BXPC3/MIAPACA2/SU86.86	up	26898939
14	MALAT1	+			+	+					BXPC3/PANC1/ASPC1/CFPAC/SW1990	up	27371730
15	ZFP91-P	+			+						BXPC3	up	27446435
16	CCDC26	+		+							ASPC1/PANC1/SW1990/BXPC3/MIAPACA2/CFPAC1	up	27470572
17	UCA1	+	+	+							PANC1/BXPC3/CAPAN1/SW1990	up	27562722
18	H19	+	+	+							COLO357/CAPAN1/MIAPACA2/ASPC1/BXPC3/PANC1/T3M4/SW1990	up	27573434
19	HOTAIR	+									BXPC3/CAPAN2/CFPAC1/PANC04.03/PANC1/SW1990	up	27594424
20	UCA1	+				+					ASPC1/BXPC3/CAPAN2/CFPAC1/HPAC/MIAPACA2/PANC1/SW1990	up	27628540

21	uc.345							+			PANC1/ASPC1/PATU 8988/BXPC3/SW1990	up	27689400
22	linc-ROR			+					GEM		PANC1/MIAPACA2/SW1 990	up	27785603
23	NEAT1	+	+	+							ASPC1/BXPC3/SW1990/ PANC1	up	27888106
24	HOTAIR	+		+	+	+					BXPC3/CFPAC1/PANC1/ L3.6PL	up	27895308
25	MALAT1	+	+	+					GEM		PANC1/BXPC3/ASPC1/S W1990/CFPAC1	up	28034748
26	CCAT1	+	+		+						PANC1/ASPC1	up	28078015
27<br>28	linc00152<br>CASC9	+				+					SW1990/BXPC3	up	28220683
29	XIST	+									PATU8988/SW1990/BXP C3/ASPC1/CFPAC1/PAN C1	up	28295543
30	ANRIL				+	+					PANC1/MIAPACA2	up	28344092
31	PVT1	+			+						PANC1	up	28355965
32	HOTAIR	+	+	+	+	+					BXPC3/CFPAC1/PANC1/ L3.6PL	up	28415631
33	HOTAIR								GEM		MIAPACA2/BXPC3/SUI T2/PANC1	up	28476883
34	linc-ROR	+	+		+	+		+			PANC1/SW1990	up	28580169
35	TUG1	+			+						SW1990/BXPC3/PATU89 88	up	28617552
36	JHDM1D-AS1						+				PANC1/ASPC1	up	28652266
37	PVT1	+			+						PANC1/ASPC1/PATU 8988/BXPC3/SW1990	up	28657147
38	MALAT1	+	+	+	+	+					PANC1/ASPC1/MIAPAC A2/BXPC3/P3/P4/P7	up	28701723
39	TUG1	+			+						PANC1/ASPC1/PATU 8988/BXPC3/SW1990	up	28813705
40	Sox2ot	+									BXPC3/PANC1	up	28867247
41	PANDAR	+	+	+							SW1990/CFPAC1/ASPC1 /PANC1/CAPAN2/BXPC3	up	28886528
42	CRNDE	+			+	+					SW1990/PANC1/CAPAN 1/JF305/BXPC3	up	28940804
43	HOTTIP							+			PANC1/SW1990	up	28947139
44	DYNC2H1-4	+			+	+		+	GEM		BXPC3-GEM/PANC1- GEM/MIAPACA2-GEM	up	28703793
45	NORAD				+	+				oxidation reaction	BXPC3/CAPAN1/PANC1/ SW1990	up	29121972
46	SNHG15	+	+	+							ASPC1/BXPC3	up	29137412
47	HOTAIR	+		+	+			+	GEM	no effect on the PANC1	PANC1/PANC1-CSCS	up	29201179
48	MALAT1	+		+	+	+					ASPC1	up	29215734
49	DUXAP10	+	+	+	+	+					BXPC3/PANC1	up	29286182
50	CCAT2	+				+					PANC1	up	29298720
51	HOXA-AS2	+	+	+							BXPC3/PANC1	up	29312501

52	H19	+	+								ASPC1/PANC1	up	29344285
53	FEZF1-AS1	+	+	+	+	+				glycolysis	CAPAN2/PANC1	up	29348628
54	XIST	+	+								BXPC3/PANC1	up	29371940
55	MALAT1	+	+	+	+	+			CDDO-Me	the loss of MALAT-1 in the p53$^{-/+}$ (heterozygote) mice increases the lifespan	MIAPACA2/PANC1	up	29389953
56	XIST	+	+		+	+					BXPC3/PANC1	up	29393501
57	linc01133	+		+							BXPC3/PANC1/SW1990	up	29458145
58	SPRY4-IT1	+	+	+	+	+					BXPC3/PANC1	up	29489909
59	UCA1	+	+	+	+	+					ASPC1	up	29500870
60	UCA1				+	+					BXPC3/PANC1/PATU8988/SW1990	up	29510195
61	SPRY4-IT1	+		+							CAPAN2/PANC1	up	29551494
62	H19				+				5-FU/abraxane		PANC1/PANC1-LUNG	up	29581580
63	SOX2OT							+		mesenchymal-like morphological change	BXPC3/CAPAN1/HS766T	up	29643475
64	HNRNPU	+			+	+					PATUT/PL45	up	29657295
65	ADPGK-AS1	+		+	+	+					PANC1/SW1990	up	29667486
66	ZEB2-AS1	+	+		+						ASPC1	up	29753015
67	MIAT	+			+	+					PATU8988	up	29772434
68	linc01121	+	+	+	+	+					Third generation of PC cells/PANC1	up	29843149
69	HOTTIP				+	+					CRL-2549™	up	29844833
70	PVT1	+			+	+					BXPC3/SW1990/PATU8988	up	29845201
71	linc00462	+	+	+	+	+					HPDE6-C7/PANC1	up	29899418
72	TUG1	+	+	+	+	+			GEM		BXPC3	up	29960845
73	BX111	+			+	+				oxidation reaction	PANC1/SW1990	up	29970904
74	PVT1	+	+	+						autophagy	CAPAN2/HPAFII/MIAPACA2/SW1990	up	30001707
75	SNHG1	+	+	+							BXPC3/PANC1	up	30087712
76	linc01296	+		+	+	+					PANC1/SW1990	up	30203487
77	AFAP1-AS1	+	+							cucurbitacin B	BXPC3/HPAC	up	30206930
78	linc00346/00578/00673	+									BXPC3/HPAC	up	30210701
79	DLX6-AS1	+			+	+					PANC1/SW1990	up	30250401
80	CUDR	+	+	+	+	+			GEM/5-FU		CFPAC1/PANC1	up	30272271
81	AFAP1-AS1	+		+	+	+					PACA2/SW1990	up	30300116
82	SUMO1P3	+			+	+					BXPC3/PANC1	up	30333879
83	PVT1	+	+	+					GEM		BXPC3/PANC1	up	30341811
84	MALAT1				+	+					ASPC1/CFPAC1	up	30352575
85	NEAT1	+			+						PANC1/MIAPACA2	up	30362505
86	DUXAP8	+	+	+							ASPC1/BXPC3/PANC1	up	30367681
87	DLEU1	+	+		+	+					CAPAN1/PANC1	up	30382579
88	HOST2	+		+					GEM		ASPC1/HS766T	up	30406400
89	H19					+		+			PANC1	up	30410672
90	HOTAIR	+		+						radiosensitivity/autophagy	ASPC1/PANC1	up	30464623
91	H19	+			+						ASPC1/PANC1	up	30474270

92	SNHG1	+			+						ASPC1/PANC1	up	30520072
93	SNHG8	+	+	+					GEM		PANC1/HS766T	up	30556854
94	UCA1			+	+	+					PANC1	up	30569514
95	ABHD11-AS1	+		+	+	+					L3.6PL/PANC1	up	30575903
96	linc01133	+			+	+					BXPC3	up	30580676
97	HULC	+		+	+	+					PANC1	up	30593805
98	TUG1	+			+	+					BXPC3/PANC1	up	30595764
99	HOTAIRM1	+	+	+	+						PANC1/SW1990	up	30613920
100	linc00958					+					BXPC3/PANC1	up	30639194
101	TP73-AS1				+	+					BXPC3/PANC1	up	30643007
102	FEZF1-AS1					+				oxidation reaction	PANC1/SW1990	up	30693518
103	linc00346	+	+	+					GEM		CAPAN1/PANC1	up	30728036
104	SNHG14	+			+	+			GEM	autophagy	SW1990	up	30737032
105	linc00994	+	+	+	+	+					ASPC1/PANC1	up	30739523
106	TUG1	+	+	+							ASPC1/BXPC3	up	30787623
107	RP11-567G11.1	+		+				+	GEM		BXPC3/PANC1	up	30802827
108	AGAP2-AS1	+	+	+	+	+					ASPC1/BXPC3/PANC1	up	30814490
109	AFAP1-AS1	+				+		+			PANC1	up	30819221
110	DLEU2	+				+					ASPC1/PACA2	up	30838724
111	UCA1	+						+			HPAFII/MPANC96/PANC1/PATU8988	up	30949406
112	SNHG16	+			+	+					ASPC1	up	30981105
113	linc01207	+		+						autophagy	MPANC96	up	30991076
114	XIST	+		+		+					PATU8988T/MIAPACA2	up	31013436
115	DLX6-AS1	+	+	+	+	+					ASPC1/BXPC3/CAPAN1/PANC1	up	31118816
116	linc00339	+			+	+					PANC1/SW1990	up	31128030
117	MIR155HG	+	+	+							PANC1/SW1990	up	31161625
118	XIST				+	+					ASPC1/PANC1	up	31163263
119	linc00473	+		+	+	+					SW1990	up	31206665
120	XIST	+			+	+					PANC1	up	31213574
121	DANCR	+	+	+	+	+					PANC1/SW1990	up	31213582
122	DANCR	+			+	+					BXPC3/MIAPACA2	up	31267381
123	AFAP1-AS1	+	+	+	+ with oridonin	+					PANC1/BXPC3(oridonin)	downregulated by oridonin	31314060
124	TP53TG1	+			+	+					PANC1/MIAPACA2(KRAS-mutant type) and BXPC3(KRAS-wide type)	up	31325400
125	HNRNPL		+		+						BXPC3/SW1990	up	31355266
126	DIO3OS	+				+					ASPC1/MIAPACA2	up	31384177
127	MACC1-AS1	+	+	+	+	+				glycolysis	PANC1/KP2	up	31391063
128	linc00346	+			+	+					BXPC3/MIAPACA2	up	31391552
129	MTA2TR	+			+	+				oxidation reaction	BXPC3/SW1990	up	31410216
130	CCHE1				+	+					CAPAN2/HPAFII	up	31423182
131	linc-RoR	+			+	+					BXPC3/PATU8988/SW1990	up	31452251
132	HOTAIR	+								glycolysis	BXPC3/CAPAN2	up	31452722

133	SNHG14	+		+		+					L3.6PL	up	31513352
134	GSTM3TV2	+		+					GEM		ASPC1-GEM/MIAPACA2-GEM	up	31514732
135	DANCR	+			+	+					PANC1/SW1990	up	31515968
136	PCAT-1				+	+					CFPAC1/PANC1	up	31539121
137	linc01420	+									BXPC3/PANC1	up	31562613
138	linc01559	+		+	+						ASPC1/BXPC3	up	31608998
139	SBF2-AS1	+	+	+	+	+			GEM		PANC1-GEM/ASPC1-GEM	up	31619579
140	HCP5	+		+	+	+			GEM	autophagy	PANC1-GEM/SW1990-GEM	up	31632071
141	linc01638				+	+					HPNE/PL45	up	31702018
142	HULC	+			+	+					PANC1 and EXO-PANC1	up	31715081
143	XLOC_006390	+			+					glycolysis/glutamate	BXPC3/CFPAC1	up	31734356
144	linc00976	+	+		+	+					PANC1/MIAPACA2	up	31747939
145	BANCR	+			+	+					PANC1/SW1990	up	31769353
146	LUCAT1	+	+		+	+					ASPC1/PANC1	up	31789465
147	EPIC1	+	+	+							HPAFII/SW1990	up	31810603
148	linc01006	+			+	+					BXPC3/PANC1	up	31827394
149	MCM3AP-AS1	+			+	+					ASPC1/PANC1	up	31830901
150	THAP9-AS1	+						+			BXPC3/CFPAC1/PANC1	up	31831555
151	SOX2OT	+	+								BXPC3/PANC1	up	31837005
152	HULC	+		+		+					BXPC3/PANC1	up	31857775
153	SNHG1	+	+								BXPC3/CAPAN1	up	31933880
154	ITGB2-AS1	+	+	+	+	+					PANC1/BXPC3	up	31957875
155	HMGA2-AS1				+						PANC1	up	32010621
156	SLC7A11-AS1							+	GEM	oxidation reaction	BXPC3-GEM/PANC1/ASPC1	up	32036249
157	NEAT1	+			+	+					BXPC3/PACA2/PANC1/SW1990	up	32064164
158	PLACT1	+			+	+					PANC1/ASPC1 (NO-KRAS/P53) AND BXPC3/CAPAN2 (KRAS/P53)	up	32085715
159	ENSG00000254041.1	+	+			+			GEM		BXPC3/PANC1/SW1990	up	32090981
160	ROCK1	+			+	+					BXPC3/HPAFII	up	32104251
161	HOTTIP	+				+					PANC1/SW1990	up	32120024
162	SNHG16				+	+					CAPAN2/PANC1	up	32141539
163	OIP5-AS1	+	+								ASPC1/PANC1	up	32157498
164	SOX2OT	+						+			BXPC3/HS766T	up	32196588
165	PVT1	+			+	+				glycolysis	HPAC	up	32201527
166	SBF2-AS1	+		+	+	+					PANC1/MP-EXO (PANC1 cells co-cultured with 200 μg OF M2 macrophage-derived exosomes for 48	up	32301277

											hours)		
167	linc285194			+	+					involved in the inhibition of PANC1 by propofol	PANC1		32303144
168	DNAH17-AS1	+		+	+	+					HS766T/SW1990	up	32351291
169	linc00162	+			+						ASPC1/PATC43/PATC50	up	32364285
170	HCP5	+			+	+					ASPC1/SW1990	up	32407143
171	SNHG12	+		+		+					CAPAN1/PANC1	up	32432698
172	SNHG6	+		+	+	+					PANC1/MIAPACA2	up	32433053
173	RUNX1-IT1	+			+	+					CFPAC1/PANC1/SW1990	up	32487998
174	PVT1									promote exosome secretion	HS766T	up	32499447
175	MVIH	+		+	+				GEM/5-FU		BXPC3/PANC1	up	32509206
176	linc00152	+			+	+					PANC1/SW1990	up	32509216
177	ZEB1-AS1	+			+	+					CAPAN1/SW1990	up	32513531
178	H19					+		+	GEM		CAPAN1/PANC1	up	32626524
179	HULC				+	+					PANC1/MIAPACA2/BXPC3/KP3/QGP1	up	32656089
180	OIP5-AS1	+			+						PANC1/BXPC3/ASPC1/CFPAC1	up	32669972
181	linc01559	+			+	+					ASPC1/BXPC3/PANC1/MIAPACA2/SW1990	up	32678071
182	linc00460	+									PANC1/SW1990	up	32724379
183	PVT1								GEM		PANC1/ASPC1/SW1990	up	32727463
184	CYTOR	+			+						ASPC1/BXPC3/PANC1/SW1990/HS766T	up	32732173
185	NT5E	+			+	+					CFPAC/COLO357/PANC1/BXPC3	up	32770626
186	linc00514	+			+	+					BXPC3/SW1990/PANC1/ASPC1/CAPAN2/MIAPACA2	up	32771045
187	linc01232				+	+					BXPC3/COLO357/CFPAC1/MIAPACA2/PANC1	up	32814086
188	PCAT6	+			+	+					CAPAN2/ASPC1/PANC1/BXPC3	up	32825947
189	PMSB8-AS1	+		+	+	+					ASPC1/BXPC3/CAPAN2/CFPAC/MIAPACA2/PANC1	up	32891166
190	MALAT1									induced by AHR	PANC1/ASPC1	up	32900487

a. Prolife: proliferation; b. Cycle: cell cycle; c. Apopt: apoptosis; d. Migra: migration; e. Invas: invasion; f. Angio: angiogenesis; g. CSC: cancer stem cell; h. GEM: gemcitabine; “+” means that the lncRNA plays a role in related cancer cell biological functions.

Table S2. Overview of mechanisms and animal studies of oncogenic lncRNAs in pancreatic cancer.

No	Lnc	Position	Location	Mechanism						Vivo functions		Pheno[g]	Reference PMID
				miRNA[c]	Target	RBPs[d]	Post-transcrip[e]	Pathways	Upstream	Grow	Meta[f]		
1	HOTAIR					PRC2				+		onco[h]	22614017
2	MALAT1							EMT				onco	25269958
3	MALAT1									+		onco	25811929
4	HOTTIP							EMT				onco	25889214
5	HOTTIP									+		onco	25912306
6	AFAP1-AS1							EMT		+		onco	25925763
7	AF339813								NUF2			onco	26045769
8	linc00675							EMT				onco	26309360
9	MIR31HG		cyto[a]	miR-193b						+		onco	26549028
10	linc-ROR			miR-145	Nanog					+		onco	26636540
11	LOC389641		cyto					EMT		+		onco	26708505
12	NUTF2P3-001			miR-3923	KRAS			KRAS		+	+	onco	26755660
13	linc-ROR							p53		+	+	onco	26898939
14	MALAT1					HuR		autophagy		+	+	onco	27371730
15	ZFP91-P											onco	27446435
16	CCDC26	8q24						PCN; Bcl2				onco	27470572
17	UCA1											onco	27562722
18	H19	11p15.5						E2F1		+		onco	27573434
19	HOTAIR					EZH2				+		onco	27594424
20	UCA1											onco	27628540
21	uc.345									+		onco	27689400
22	linc-ROR			miR-124	PTBP1			autophagy				onco	27785603
23	NEAT1			miR-506-3p								onco	27888106
24	HOTAIR									+		onco	27895308
25	MALAT1								miR-216a			onco	28034748
26	CCAT1								c-Myc			onco	28078015
27	linc00152											onco	28220683
28	CASC9												
29	XIST			miR-133a	EGFR			AKT				onco	28295543
30	ANRIL							ATM-E2F1				onco	28344092
31	PVT1	8q24.21						p21; ZEB1				onco	28355965
32	HOTAIR			miR-613	Notch3			Notch3				onco	28415631
33	HOTAIR					EZH2						onco	28476883
34	linc-ROR			mutiple				TGF-β/Smad				onco	28580169
35	TUG1											onco	28617552
36	JHDM1D-AS1		cyto; nucl									onco	28652266
37	PVT1	8q24.21	cyto	miR-448	SERBP1							onco	28657147
38	MALAT1		nucl[b]	miR-217	KRAS							onco	28701723
39	TUG1		cyto	miR-382	EZH2			EMT				onco	28813705
40	Sox2ot							Sox2	YY1	+		onco	28867247
41	PANDAR	6p21.2								+		onco	28886528
42	CRNDE			miR-384	IRS1					+	+	onco	28940804

43	HOTTIP					WDR5	Wnt/β-catenin		+		onco	28947139
44	DYNC2H1-4		cyto	miR-145-5p	MMP3		EMT				onco	28703793
45	NORAD			miR-125a-3p	RhoA		EMT			+	onco	29121972
46	SNHG15	7p13	cyto			EZH2/SUZ12	p15/KLF2		+		onco	29137412
47	HOTAIR										onco	29201179
48	MALAT1						LATS1; YAP1		+		onco	29215734
49	DUXAP10					EZH2/LSD1			+		onco	29286182
50	CCAT2	8q24				KRAS	MEK/ERK		+		onco	29298720
51	HOXA-AS2					EZH2/LSD1			+		onco	29312501
52	H19	11p15.5		miR-675	E2F1				+		onco	29344285
53	FEZF1-AS1		cyto	miR-107	ZNF312B				+		onco	29348628
54	XIST	X		miR-140/124	iASPP		CCND1; CDK1; p21	iASPP/p73	+		onco	29371940
55	MALAT1					APAF1; NDRG1		SP1/SP3/SP4	+		onco	29389953
56	XIST	X		miR-34a-5p					+		onco	29393501
57	linc01133						CCNG1	C/EBPβ	+		onco	29458145
58	SPRY4-IT1						CDC20				onco	29489909
59	UCA1	19p13.12	cyto	miR-96	FOXO3						onco	29500870
60	UCA1	19p13.12				Hippo (MOB1/Lats1/ YAP)	YAP	YAP			onco	29510195
61	SPRY4-IT1								+		onco	29551494
62	H19									+	onco	29581580
63	SOX2OT		cyto	miR-200s	SOX2		EMT		+	+	onco	29643475
64	HNRNPU										onco	29657295
65	ADPGK-AS1			miR-205	ZEB1		EMT		+		onco	29667486
66	ZEB2-AS1		cyto	miR-204	HMGB1		EMT		+		onco	29753015
67	MIAT			miR-133					+		onco	29772434
68	linc01121					GLP	cAMP/PKA				onco	29843149
69	HOTTIP						mGluR1				onco	29844833
70	PVT1						TGF-β/Smad/EMT				onco	29845201
71	linc00462			miR-665	TGFBR1/2		SMAD2/3		+	+	onco	29899418
72	TUG1						ERK				onco	29960845
73	BX111	10:31759 883-31760177	nucl			YB1	ZEB1/EMT	HIF-1a	+	+	onco	29970904
74	PVT1	8q24	cyto (mainly) and nucl	miR-20a-5p	ULK1				+		onco	30001707
75	SNHG1	11q12.3					PI3K/AKT		+		onco	30087712
76	linc01296	14q11.2					EMT				onco	30203487
77	AFAP1-AS1			miR-146b-5p	EGFR		p-EGFR/p-AKT				onco	30206930
78	linc00346/005 78/00673										onco	30210701
79	DLX6-AS1			miR-181b	ZEB2		EMT		+	+	onco	30250401
80	CUDR						EMT; AKT/ERK		+		onco	30272271
81	AFAP1-AS1			miR-133a-5p	IGF1R		EMT		+		onco	30300116

82	SUMO1P3						EMT				onco	30333879
83	PVT1	8q24		miR-1207-5p; miR-1207-3p	SRC; RhoA		suppress the generation of mature miRNAs from precursor miRNAs through Drosha/DGCR8				onco	30341811
84	MALAT1			miR-200c-3p	ZEB1			miR-200c-3p			onco	30352575
85	NEAT1			miR-302a-3p	RELA			RELA			onco	30362505
86	DUXAP8					EZH2/LSD1	CDKN1A/KLF2		+		onco	30367681
87	DLEU1	13q14.3	cyto	miR-381	CXCR4				+		onco	30382579
88	HOST2										onco	30406400
89	H19						CD24; Integrin				onco	30410672
90	HOTAIR										onco	30464623
91	H19			miR-194	PFTK1		Wnt pathway				onco	30474270
92	SNHG1						Notch pathway				onco	30520072
93	SNHG8										onco	30556854
94	UCA1			miR-107	ITGA2		Focal adhesion pathway				onco	30569514
95	ABHD11-AS1	7q11.23					EMT; PI3K/Akt				onco	30575903
96	linc01133		nucl			DKK1	Wnt-5a/MMP-7/β-catenin		+	+	onco	30580676
97	HULC			miR-15a			PI3K/Akt				onco	30593805
98	TUG1			miR-29c			ITGB1/MMP2/MMP9/EMT		+		onco	30595764
99	HOTAIRM1	7p15					EMT				onco	30613920
100	linc00958	11	cyto	miR-330-5p	PAX8		EMT		+	+	onco	30639194
101	TP73-AS1	1p36.32		miR-141	BDH2						onco	30643007
102	FEZF1-AS1			miR-142-3p; miR-133a	HIF-1a; EGFR						onco	30693518
103	linc00346			miR-188-3p	BRD4				+		onco	30728036
104	SNHG14			miR-101							onco	30737032
105	linc00994			miR-765-3p	RUNX2				+		onco	30739523
106	TUG1	GRCh38.p7	nucl			EZH2	RND3/MT2A		+		onco	30787623
107	RP11-567G11.1	3					Notch pathway (Jagged1/HES1/HES5/Math1)				onco	30802827
108	AGAP2-AS1		nucl			EZH2	ANKRD1/ANGPTL4	RREB1	+		onco	30814490
109	AFAP1-AS1		cyto	miR-384	ACVR1				+		onco	30819221
110	DLEU2	13q14.3		miR-455	SMAD2			miR-15a/miR-16			onco	30838724
111	UCA1	19q13.12		miR-590-3p	KRAS	hnRNPA2B1	KRAS		+		onco	30949406
112	SNHG16			miR-218-5p	HMGB1				+		onco	30981105
113	linc01207	4q32		miR-143-5p	AGR2						onco	30991076
114	XIST	X		miR-34a-5p			YAP/EGFR/TGF-β1/EMT				onco	31013436

115	DLX6-AS1	7q21.3		miR-497-5p	FZD4/FZD6		Wnt/β-catenin		+	+	onco	31118816
116	linc00339			miR-497-5p	IGF1R						onco	31128030
117	MIR155HG			miR-802							onco	31161625
118	XIST	X		miR-429	ZEB1		EMT				onco	31163263
119	linc00473		cyto	miR-195-5p	PD-L1		inhibit the activation of CD8+ T cells				onco	31206665
120	XIST	X		miR-141-3p	TGF-β2						onco	31213574
121	DANCR			miR-214-5p	E2F2				+		onco	31213582
122	DANCR			miR-135a-5p	NLRP3		EMT		+		onco	31267381
123	AFAP1-AS1								+		onco	31314060
124	TP53TG1	12	cyto	miR-96	KRAS						onco	31325400
125	HNRNPL					PTBP1	EMT				onco	31355266
126	DIO3OS			miR-122	ALDOA						onco	31384177
127	MACC1-AS1		cyto			PAX8	NOTCH1		+	+	onco	31391063
128	linc00346					CTCF	MYC		+		onco	31391552
129	MTA2TR	11	nucl			ATF3	MTA2 (ATF3 promote transcription)/HIF-1a (deacetylation)	HIF-1a	+	+	onco	31410216
130	CCHE1					ROCK1					onco	31423182
131	linc-RoR						Hippo/Yap/EMT				onco	31452251
132	HOTAIR					HK2					onco	31452722
133	SNHG14		cyto	miR-163	ANXA2				+		onco	31513352
134	GSTM3TV2		cyto	miR-let-7	LAT2/OLR1				+		onco	31514732
135	DANCR	4q12		miR-33b	MMP16		EMT				onco	31515968
136	PCAT-1					RBM5					onco	31539121
137	linc01420		cyto(40%) and nucl(60%)	miR-494-3p	MYC	MYC	EMT; KRAS/EMT		+		onco	31562613
138	linc01559		cyto	miR-607	YAP	YAP			+		onco	31608998
139	SBF2-AS1		cyto	miR-142-3p	TWF1		EMT				onco	31619579
140	HCP5			miR-214-3p	HDGF				+		onco	31632071
141	linc01638						TGF-β1				onco	31702018
142	HULC						EMT	TGF-β/miR-133b	+		onco	31715081
143	XLOC_006390					c-MYC	GDH1/aKG		+		onco	31734356
144	linc00976		cyto	miR-137	OTUD7B		EGFR (deubiquitinate)/MAPK(MEK/ERK/p38/SAPK/JUK)		+	+	onco	31747939
145	BANCR	9		miR-195-5p			Wnt/β-catenin				onco	31769353
146	LUCAT1	5		miR-539	EMT				+		onco	31789465
147	EPIC1					YAP	CDC20/CDK4/CCNDA1				onco	31810603
148	linc01006		cyto (mainly)	miR-2682-5p	HOXB8			HOXB8	+	+	onco	31827394

			and nucl										
149	MCM3AP-AS1			miR-138-5p	FOXK1			MMP		+		onco	31830901
150	THAP9-AS1		cyto; nucl	miR-484	YAP	YAP		CTGF, CYR61	TEAD1/YAP	+		onco	31831555
151	SOX2OT		nucl			FUS		CCND1/p27		+		onco	31837005
152	HULC							Wnt/β-catenin				onco	31857775
153	SNHG1	11q12.3		miR-195	CCND1			p21		+		onco	31933880
154	ITGB2-AS1		cyto	miR-4319	RAF1			MEK/ERK		+	+	onco	31957875
155	HMGA2-AS1					HMGB2		EMT				onco	32010621
156	SLC7A11-AS1		nucl			β-TRCP1	NRF2/GCLM/ HMOX1	SKP1/CUL1/RBX1 /E2; prevent NRF2 ubiquitination and β-catenin		+		onco	32036249
157	NEAT1					IGF2BP1	ELF3	ELF3		+	+	onco	32064164
158	PLACT1		cyto; nucl			hnRNPA1-H3K27me3-IkBa; IkBa promoter		E2F1	E2F1	+	+	onco	32085715
159	ENSG00000254041.1					SOX4		EMT				onco	32090981
160	ROCK1							ROCK1				onco	32104251
161	HOTTIP					HOXA13	WDR5-MLL1/ HOXA13	CHI3L1, CLIC5, CYP26B1, UCP2, CYB5R2, SULT1A1, KIF26A, SLC1A4, TSC22D1	miR-497	+		onco	32120024
162	SNHG16			miR-200a-3p								onco	32141539
163	OIP5-AS1			miR-342-3p	AGR2			AKT/ERK		+		onco	32157498
164	SOX2OT			miR-200a/200b/141	DEK					+		onco	32196588
165	PVT1			miR-519d-3p	HIF-1a					+		onco	32201527
166	SBF2-AS1		cyto (mainly) and nucl	miR-122-5p	XIPA					+		onco	32301277
167	linc285194			miR-34a				E-cadherin				suppr	32303144
168	DNAH17-AS1		cyto (mainly) and nucl	miR-432-5p	PPME1							onco	32351291
169	linc00162		cyto						p65	+		onco	32364285
170	HCP5			miR-140-5p	CDK8					+		onco	32407143
171	SNHG12	1		miR-320b				EMT				onco	32432698
172	SNHG6			miR-26a-5p	FUBP1			EMT		+		onco	32433053
173	RUNX1-IT1		nucl			RUNX1	RUNX1				+	onco	32487998
174	PVT1		cyto			RAB7						onco	32499447
175	MVIH											onco	32509206
176	linc00152	2p11.2		miR-150	ZEB1			EMT		+		onco	32509216
177	ZEB1-AS1			miR-505-3p	TRIB2							onco	32513531

178	H19	11p15.5		miR-675-3p	SOCS5			EMT		+		onco	32626524
179	HULC							EMT	miR-622			onco	32656089
180	OIP5-AS1			miR-429	FOXD1			EMT		+		onco	32669972
181	linc01559		cyto	miR-1343-3p	RAF1			MEK/ERK		+		onco	32678071
182	linc00460	13: 106376564-106378595										onco	32724379
183	PVT1	8q24	cyto; nucl	miR-619-5p	Pygo2; ATG14	ATG14		autophagy				onco	32727463
184	CYTOR			miR-205-5p	CDK6					+		onco	32732173
185	NT5E	6q14.3								+		onco	32770626
186	linc00514		cyto	miR-28-5p	Rap1b					+	+	onco	32771045
187	linc01232	13q32	nucl				HNRNPA2B1	A-Raf/MAPK			+	onco	32814086
188	PCAT6			miR-185-5p	CBX2							onco	32825947
189	PMSB8-AS1		cyto	miR-382-3p	STAT1			EMT		+	+	onco	32891166
190	MALAT1					EZH2						onco	32900487

a. cyto: cytoplasm; b. nucl: nucleus; c. miRNAs: microRNAs; d. RBPs: RNA binding proteins; e. Post-transcrip; post-transcription; f. Meta: metastasis; g. Pheno: phenomenon; h. onco: oncogene; "+" means that the lncRNA plays a role in tumor growth or metastasis.

Table S3. Overview of clinicopathological significance of oncogenic lncRNAs in pancreatic cancer.

No	Lnc	Source	Express	Cut-off	Variables								Reference PMID
					Size	Differentiation	T	Lymphatic (N)	Distance (M)	TNM	Vessel invasion	Other	
1	MALAT1	tissue	up	0.1035	+					+		depth of invasion	24815433
2	MALAT1	tissue	up										25269958
3	HOTTIP	tissue	up										25889214
4	HOTTIP	tissue	up					+				nerve invasion	25925763
5	linc00675	tissue	up					+				nerve invasion	26309360
	HOTTIP-005	tissue	up			+		+					
	XLOC_006390	tissue	up									nerve invasion	
6	RP11-567G11.1	tissue	up				+	+					26447755
	HDRF	plasma	up	11.4									
	RDRF	plasma	up	11.925									
7	MIR31HG	tissue	up										26549028
8	linc-ROR	tissue	up		+								26636540
9	HOTAIRM1	tissue	up										26676849
10	LOC389641	tissue	up					+		+			26708505
11	NUTF2P3-001	tissue	up		+	+		+	+	+			26755660
12	linc-ROR	tissue	up										26898939
13	HOTAIR	saliva	up	2.1689									27028998
	PVT1	saliva	up	1.2399									
14	MALAT1	tissue	up						+	+			27371730
15	CCDC26	tissue	up		+								27470572
16	UCA1	tissue	up		+					+		depth of invasion	27562722
17	H19	tissue	up			+							27573434
	CRNDE	tissue	up										
	NR_036488	tissue	up										
18	ENSG00000244649	tissue	up										27628540
	AFAP1-AS1	tissue	up		+								
	UCA1	tissue	up				+						
19	uc.345	tissue	up							+		depth of invasion	27689400
20	linc-ROR	tissue	up										27785603
21	NEAT1	tissue	up							+		depth of invasion	27888106
22	HOTAIR	tissue	up										27895308
23	MALAT1	tissue	up										28034748
24	CCAT1	tissue	up										28078015
25	XIST	tissue	up		+					+			28295543
26	PVT1	tissue	up					+		+			28355965
27	HOTAIR	tissue	up										28415631
28	linc-ROR	tissue	up										28580169
29	TUG1	tissue	up										28617552
30	PVT1	tissue	up		+	+			+	+	+		28657147
31	TUG1	tissue	up		+	+			+	+	+		28813705
32	Sox2ot	tissue	up	0.121						+			28867247
33	PANDAR	tissue	up							+	+		28886528

34	RP11-263F15.1	tissue	up			+						28928863
35	CRNDE	tissue	up		+	+		+		+		28940804
36	HOTTIP	tissue	up									28947139
37	DYNC2H1-4	tissue	up									28703793
38	NORAD	tissue	up									29121972
39	SNHG15	tissue	up	MEL[a]	+			+		+		29137412
40	MALAT1	tissue	up									29215734
41	MALAT1/HOTTIP /PVT1	plasma	up								patients with low expression had a better prognosis after GEM treatment	29221115
42	DUXAP10	tissue	up	MEL				+		+		29286182
43	CCAT2	tissue	up			+	+					29298720
44	HOXA-AS2	tissue	up									29312501
45	FEZF1-AS1	tissue	up	MEL		+				+	perineural invasion	29348628
46	XIST	tissue	up	MEL	+					+		29371940
47	XIST	tissue	up						+	+		29393501
48	linc01133	tissue	up		+	+	+			+	mutation count	29458145
49	UCA1	tissue	up									29500870
50	UCA1	BADE A	up							+	pathologic type	29510195
51	SPRY4-IT1	tissue	up			+	+					29551494
52	H19	tissue	up			+						29581580
53	SOX2OT	plasma	up							+	decreased after operation	29643475
54	HNRNPU	tissue	up								high expressed in bad prognosis	29657295
55	ZEB2-AS1	tissue	up									29753015
56	MIAT	tissue	up	5.8								29772434
57	HOTTIP	tissue	up		+				+			29844833
58	PVT1	tissue	up									29845201
59	linc00462	tissue	up		+	+			+	+		29899418
60	TUG1	tissue	up									29960845
61	BX111	tissue	up						+			29970904
62	PVT1	tissue	up				+	+		+		30001707
63	SNHG1	tissue	up		+					+		30087712
64	linc01296	tissue	up	2.47				+		+		30203487
	linc00346	tissue	up					+		+		
65	linc00346/00578/0 linc0673	plasma	up									30210701
	linc00578	tissue	up					+	+	+		
	linc00673	tissue	up						+	+		
66	DLX6-AS1	tissue	up		+			+		+		30250401
67	CUDR	tissue	up									30272271
68	SNHG15	tissue	up	MV[b]		+		+		+		30280769
69	SNHG15	plasma	up									30280769
70	AFAP1-AS1	tissue	up		+			+		+		30300116
71	SUMO1P3	tissue	up	MV	+			+		+		30333879
72	MALAT1	tissue	up	MEL				+		+		30352575
73	NEAT1	tissue	up	MEL				+		+		30362505
74	DUXAP8	tissue	up	MEL	+					+		30367681

84	DLEU1	tissue	up	MV		+				+	perineural invasion	30382579
85	HOTAIR	tissue	up				+	+			radiosensitivity	30464623
86	H19	tissue	up	MEL					+	+		30474270
78	SNHG8	tissue	up	MEL		+				+		30556854
79	UCA1	tissue	up									30569514
80	ABHD11-AS1	tissue	up			+			+	+		30575903
81	HULC	tissue	up									30593805
82	TUG1	tissue	up	MV		+		+		+		30595764
83	HOTAIRM1	tissue	up								mutations of KRAS	30613920
84	TP73-AS1	tissue	up					+		+		30643007
85	FEZF1-AS1	tissue	up	MEL	+			+		+		30693518
86	SNHG14	tissue	up									30737032
87	linc00994	tissue	up									30739523
88	TUG1	tissue	up	MEL	+					+		30787623
89	RP11-567G11.1	tissue	up			+						30802827
90	AGAP2-AS1	tissue	up	MEL	+			+		+		30814490
91	AFAP1-AS1	tissue	up		+			+		+		30819221
92	DLEU2	tissue	up									30838724
93	UCA1	tissue	up									30949406
94	SNHG16	tissue	up	MEL		+		+		+		30981105
95	linc01207	tissue	up									30991076
96	XIST	tissue	up								low in male	31013436
97	DLX6-AS1	tissue	up									31118816
98	linc00339	tissue	up									31128030
99	MIR155HG	tissue	up	MEL			+			+		31161625
100	XIST	tissue	up						+			31163263
101	linc00473	tissue	up									31206665
102	XIST	tissue	up									31213574
103	DANCR	tissue	up	MEL	+			+		+		31213582
104	DANCR	tissue	up	MEL				+		+		31267381
105	TP53TG1	tissue	up									31325400
106	ABHD11-AS1	plasma	up								increased progressively in the healthy/CP/PC(I/II) groups	31333792
107	HNRNPL	tissue	up							+	gender	31355266
108	DIO3OS	tissue	up									31384177
109	MACC1-AS1	tissue	up							+		31391063
110	linc00346	tissue	up									31391552
111	MTA2TR	tissue	up		+	+		+	+	+		31410216
112	CCHE1	plasma	up						+			31423182
113	HOTAIR	tissue	up									31452722
114	HOTAIR	plasma	up							+		31452722
115	SNHG14	tissue	up			+		+		+		31513352
116	GSTM3TV2	tissue	up					+		+		31514732
117	DANCR	tissue	up									31515968
118	PCAT-1	tissue	up									31539121
119	linc01420	tissue	up									31562613
120	linc01559	tissue	up	MEL								31608998
121	SBF2-AS1	tissue	up	3.09		+		+		+		31619579

122	HCP5	tissue	up										31632071
123	linc01638	tissue	up										31702018
124	linc01638	plasma	up										31702018
125	HULC	plasma exosomes	up										31715081
126	XLOC_006390	tissue	up										31734356
127	linc00976	tissue	up		+			+	+		+	perineural invasion	31747939
128	BANCR	tissue	up						+	+			31769353
129	LUCAT1	tissue	up		+			+					31789465
130	EPIC1	tissue	up		+			+		+			31810603
131	linc01006	tissue	up										31827394
132	MCM3AP-AS1	tissue	up	MEL	+			+		+			31830901
133	THAP9-AS1	tissue	up										31831555
134	HULC	plasma	up		+		+		+		+		31857775
135	UFC1	plasma	up					+	+	+			31933809
136	SNHG1	tissue	up	MV	+					+			31933880
137	ITGB2-AS1	tissue	up			+		+		+			31957875
138	SLC7A11-AS1	tissue	up										32036249
139	NEAT1	tissue	up	MEL				+	+	+			32064164
140	MALAT1/CRNDE	serum exosomes	up (PDAC III/IV than health or IPMN)										32071328
141	PLACT1	tissue	up	MEL				+		+			32085715
142	ENSG00000254041.1	tissue	up	MEL									32090981
143	ROCK1	plasma	up										32104251
144	HOTTIP	tissue	up										32120024
145	SNHG16	tissue	up										32141539
146	OIP5-AS1	tissue	up					+		+			32157498
147	SOX2OT	tissue	up	MEL						+		CD44	32196588
148	PVT1	tissue	up	MEL				+					32201527
149	DNAH17-AS1	tissue	up			+		+		+			32351291
150	HCP5	tissue	up	MEL	+	+		+		+			32407143
151	SNHG12	tissue	up						+				32432698
152	SNHG6	tissue	up										32433053
153	RUNX1-IT1	tissue and microarray	up	ISH staining score		+		+		+			32487998
154	MVIH	tissue	up		+			+		+			32509206
155	linc00152	tissue	up										32509216
156	ZEB1-AS1	tissue	up							+			32513531
157	CASC8	tissue	up										32582694
158	OIP5-AS1	tissue	up		+				+	+			32669972

159	linc01559	tissue	up	+	+				32678071
160	linc00460	tissue	up	+					32724379
161	CYTOR	tissue	up						32732173
162	NT5E	tissue	up		+		+		32770626
163	linc00514	tissue	up	+	+		+		32771045
164	linc01232	tissue	up					nerve invasion	32814086
165	PCAT6	tissue	up		+		+		32825947
166	PMSB8-AS1	tissue	up	+		+	+	nerve invasion	32891166

a. MEL: median expression level; b. MV: mean value; "+" means that the lncRNA plays a role in related PC clinicopathological features.

Table S4. Overview of prognostic and diagnostic significance of oncogenic lncRNAs in pancreatic cancer.

No	Lnc	Source	Expression	Detection method	AUC[a]	Survival	Prognostic biomarker	Reference PMID
1	MALAT1	tissue	up	qRT-PCR	0.69	DFS[b]	+	24815433
2	MALAT1	tissue	up	qRT-PCR				25269958
3	HOTTIP	tissue	up	qRT-PCR				25889214
4	HOTTIP	tissue	up	qRT-PCR		OS[c]		25925763
5	linc00675	tissue	up	qRT-PCR		OS	+	26309360
	HOTTIP-005	tissue	up	qRT-PCR		OS	+	
	XLOC_006390	tissue	up	qRT-PCR				
6	RP11-567G11.1	tissue	up	qRT-PCR		OS	+	26447755
	HDRF	plasma	up	qRT-PCR	0.857	OS	+	
	RDRF	plasma	up	qRT-PCR	0.770	OS	+	
7	MIR31HG	tissue	up	qRT-PCR				26549028
8	linc-ROR	tissue	up	qRT-PCR		OS		26636540
9	HOTAIRM1	tissue	up	qRT-PCR				26676849
10	LOC389641	tissue	up	qRT-PCR		OS	+	26708505
11	NUTF2P3-001	tissue	up	qRT-PCR		OS		26755660
12	linc-ROR	tissue	up	qRT-PCR				26898939
13	HOTAIR	saliva	up	qRT-PCR	0.88			27028998
	PVT1	saliva	up	qRT-PCR	0.87			
14	MALAT1	tissue	up	qRT-PCR		OS	+	27371730
15	CCDC26	tissue	up	qRT-PCR	0.663	OS	+	27470572
16	UCA1	tissue	up	qRT-PCR		OS	+	27562722
17	H19	tissue	up	qRT-PCR				27573434
	CRNDE	tissue	up	qRT-PCR	0.589			
	NR_036488	tissue	up	qRT-PCR	0.624			
18	ENSG00000244649	tissue	up	qRT-PCR	0.622			27628540
	AFAP1-AS1	tissue	up	qRT-PCR	0.733	OS		
	UCA1	tissue	up	qRT-PCR	0.715	OS	+	
19	uc.345	tissue	up	qRT-PCR		OS	+	27689400
20	linc-ROR	tissue	up	qRT-PCR				27785603
21	NEAT1	tissue	up	qRT-PCR		OS		27888106
22	HOTAIR	tissue	up	qRT-PCR				27895308
23	MALAT1	tissue	up	qRT-PCR				28034748
24	CCAT1	tissue	up	qRT-PCR				28078015
25	XIST	tissue	up	qRT-PCR		OS	+	28295543
26	PVT1	tissue	up	qRT-PCR				28355965
27	HOTAIR	tissue	up	qRT-PCR				28415631
28	linc-ROR	tissue	up	qRT-PCR		OS		28580169
29	TUG1	tissue	up	qRT-PCR				28617552
30	PVT1	tissue	up	qRT-PCR		OS		28657147
31	TUG1	tissue	up	qRT-PCR		OS		28813705
32	Sox2ot	tissue	up	qRT-PCR	0.784	OS		28867247
33	PANDAR	tissue	up	qRT-PCR				28886528
34	RP11-263F15.1	tissue	up	qRT-PCR	0.843	OS		28928863
35	CRNDE	tissue	up	qRT-PCR		OS		28940804
36	HOTTIP	tissue	up	qRT-PCR		OS/DFS		28947139

37	DYNC2H1-4	tissue	up	qRT-PCR				28703793
38	NORAD	tissue	up	qRT-PCR		OS/DFS		29121972
39	SNHG15	tissue	up	qRT-PCR				29137412
40	MALAT1	tissue	up	qRT-PCR				29215734
41	MALAT1/HOTTIP/PVT1	plasma	up	qRT-PCR				29221115
42	DUXAP10	tissue	up	qRT-PCR				29286182
43	CCAT2	tissue	up	qRT-PCR		OS		29298720
44	HOXA-AS2	tissue	up	qRT-PCR				29312501
45	FEZF1-AS1	tissue	up	ISH and qRT-PCR		OS	+	29348628
46	XIST	tissue	up	qRT-PCR		OS/DFS	+	29371940
47	XIST	tissue	up	qRT-PCR		OS		29393501
48	linc01133	tissue	up	qRT-PCR		OS/DFS		29458145
49	UCA1	tissue	up	qRT-PCR		OS		29500870
50	UCA1	BADEA	up	ISH and qRT-PCR		OS		29510195
51	SPRY4-IT1	tissue	up	qRT-PCR		OS	+	29551494
52	H19	tissue	up	ISH				29581580
53	SOX2OT	plasma	up	qRT-PCR		OS	+	29643475
54	HNRNPU	tissue	up	qRT-PCR				29657295
55	ZEB2-AS1	tissue	up	qRT-PCR		OS/DFS		29753015
56	MIAT	tissue	up	qRT-PCR		OS		29772434
57	HOTTIP	tissue	up	qRT-PCR		OS		29844833
58	PVT1	tissue	up	qRT-PCR				29845201
59	linc00462	tissue	up	qRT-PCR			+	29899418
60	TUG1	tissue	up	qRT-PCR				29960845
61	BX111	tissue	up	qRT-PCR		OS (resected and non-resected)		29970904
62	PVT1	tissue	up	ISH and qRT-PCR		OS		30001707
63	SNHG1	tissue	up	qRT-PCR				30087712
64	linc01296	tissue	up	qRT-PCR		OS	+	30203487
65	linc00346	tissue	up	qRT-PCR	0.7073	OS		30210701
	linc00346/00578/00673	plasma	up	qRT-PCR				
	linc00578	tissue	up	qRT-PCR	0.7837	OS		
	linc00673	tissue	up	qRT-PCR	0.6093	OS		
66	DLX6-AS1	tissue	up	qRT-PCR				30250401
67	CUDR	tissue	up	qRT-PCR				30272271
68	SNHG15	tissue	up	qRT-PCR	0.785	OS	+	30280769
69	SNHG15	plasma	up	qRT-PCR	0.727			30280769
70	AFAP1-AS1	tissue	up	qRT-PCR		OS		30300116
71	SUMO1P3	tissue	up	qRT-PCR		OS	+	30333879
72	MALAT1	tissue	up	qRT-PCR		OS	+	30352575
73	NEAT1	tissue	up	qRT-PCR		OS	+	30362505
74	DUXAP8	tissue	up	qRT-PCR		OS		30367681
84	DLEU1	tissue	up	FISH and qRT-PCR		OS	+	30382579
85	HOTAIR	tissue	up	TCGA		OS		30464623
86	H19	tissue	up	qRT-PCR		OS	+	30474270
78	SNHG8	tissue	up	qRT-PCR		OS		30556854
79	UCA1	tissue	up	qRT-PCR		OS		30569514
80	ABHD11-AS1	tissue	up	qRT-PCR		OS/DFS	+	30575903

81	HULC	tissue	up	qRT-PCR				30593805
82	TUG1	tissue	up	qRT-PCR		OS		30595764
83	HOTAIRM1	tissue	up	qRT-PCR				30613920
84	TP73-AS1	tissue	up	qRT-PCR		OS		30643007
85	FEZF1-AS1	tissue	up	qRT-PCR		OS	+	30693518
86	SNHG14	tissue	up	qRT-PCR				30737032
87	linc00994	tissue	up	qRT-PCR				30739523
88	TUG1	tissue	up	qRT-PCR		OS		30787623
89	RP11-567G11.1	tissue	up	ISH and qRT-PCR				30802827
90	AGAP2-AS1	tissue	up	qRT-PCR		OS		30814490
91	AFAP1-AS1	tissue	up	ISH and qRT-PCR				30819221
92	DLEU2	tissue	up	Oncomine/UALCAN		OS		30838724
93	UCA1	tissue	up	TCGA		OS		30949406
94	SNHG16	tissue	up	qRT-PCR		OS		30981105
95	linc01207	tissue	up	qRT-PCR				30991076
96	XIST	tissue	up	qRT-PCR				31013436
97	DLX6-AS1	tissue	up	qRT-PCR		OS		31118816
98	linc00339	tissue	up	qRT-PCR				31128030
99	MIR155HG	tissue	up	qRT-PCR		OS		31161625
100	XIST	tissue	up	qRT-PCR				31163263
101	linc00473	tissue	up	qRT-PCR				31206665
102	XIST	tissue	up	qRT-PCR				31213574
103	DANCR	tissue	up	qRT-PCR		OS		31213582
104	DANCR	tissue	up	qRT-PCR		OS	+	31267381
105	TP53TG1	tissue	up	qRT-PCR				31325400
106	ABHD11-AS1	plasma	up	qRT-PCR	0.921(combination with CA199 to early PC)	OS		31333792
107	HNRNPL	tissue	up	TCGA		OS	+	31355266
108	DIO3OS	tissue	up	TCGA		OS		31384177
109	MACC1-AS1	tissue	up	qRT-PCR		OS/DFS	+	31391063
110	linc00346	tissue	up	GSE28735		OS		31391552
111	MTA2TR	tissue	up	RNA-FISH and qRT-PCR		OS		31410216
112	CCHE1	plasma	up	qRT-PCR	0.8895			31423182
113	HOTAIR	tissue	up	qRT-PCR				31452722
114	HOTAIR	plasma	up	qRT-PCR	0.9329	OS		31452722
115	SNHG14	tissue	up	qRT-PCR				31513352
116	GSTM3TV2	tissue	up	ISH		OS	+	31514732
117	DANCR	tissue	up	qRT-PCR				31515968
118	PCAT-1	tissue	up	qRT-PCR				31539121
119	linc01420	tissue	up	TCGA				31562613
120	linc01559	tissue	up	TCGA		OS		31608998
121	SBF2-AS1	tissue	up	qRT-PCR		OS		31619579
122	HCP5	tissue	up	qRT-PCR		OS		31632071
123	linc01638	tissue	up	qRT-PCR				31702018
124	linc01638	plasma	up	qRT-PCR	0.876			31702018
125	HULC	plasma exosomes	up	qRT-PCR	0.92			31715081

126	XLOC_006390	tissue	up	qRT-PCR				31734356
127	linc00976	tissue	up	ISH and qRT-PCR		OS		31747939
128	BANCR	tissue	up	qRT-PCR				31769353
129	LUCAT1	tissue	up	ISH and qRT-PCR				31789465
130	EPIC1	tissue	up	qRT-PCR				31810603
131	linc01006	tissue	up	qRT-PCR				31827394
132	MCM3AP-AS1	tissue	up	ISH and qRT-PCR		OS	+	31830901
133	THAP9-AS1	tissue	up	qRT-PCR		OS	+	31831555
134	HULC	plasma	up	qRT-PCR	0.856	OS	+	31857775
135	UFC1	plasma	up	qRT-PCR	0.81	OS/DFS	+	31933809
136	SNHG1	tissue	up	qRT-PCR		OS		31933880
137	ITGB2-AS1	tissue	up	ISH and qRT-PCR		OS		31957875
138	SLC7A11-AS1	tissue	up	qRT-PCR		OS		32036249
139	NEAT1	tissue	up	ISH and qRT-PCR		OS		32064164
140	MALAT1/CRNDE	serum exosomes	up (PDAC III/IV than health or IPMN)	qRT-PCR				32071328
141	PLACT1	tissue	up	ISH and qRT-PCR		OS/DFS	+	32085715
142	ENSG00000254041.1	tissue	up	qRT-PCR	0.71 (0.78 combine AJCC stage)	OS	+	32090981
143	ROCK1	plasma	up	qRT-PCR	0.8488			32104251
144	HOTTIP	tissue	up	qRT-PCR				32120024
145	SNHG16	tissue	up	qRT-PCR				32141539
146	OIP5-AS1	tissue	up	qRT-PCR				32157498
147	SOX2OT	tissue	up	qRT-PCR		OS		32196588
148	PVT1	tissue	up	qRT-PCR		OS		32201527
149	DNAH17-AS1	tissue	up	qRT-PCR		OS/DFS		32351291
150	HCP5	tissue	up	qRT-PCR		OS		32407143
151	SNHG12	tissue	up	qRT-PCR				32432698
152	SNHG6	tissue	up	qRT-PCR				32433053
153	RUNX1-IT1	tissue and microarray	up	ISH and qRT-PCR		OS	+	32487998
154	MVIH	tissue	up	qRT-PCR		OS	+	32509206
155	linc00152	tissue	up	qRT-PCR				32509216
156	ZEB1-AS1	tissue	up	qRT-PCR		OS		32513531
157	CASC8	tissue	up	qRT-PCR				32582694
158	OIP5-AS1	tissue	up	qRT-PCR		OS	+	32669972
159	linc01559	tissue	up	qRT-PCR		OS		32678071
160	linc00460	tissue	up	qRT-PCR		DFS	+	32724379
161	CYTOR	tissue	up	qRT-PCR		OS		32732173
162	NT5E	tissue	up	qRT-PCR		OS/PFS[d]	+	32770626
163	linc00514	tissue	up	qRT-PCR		OS		32771045
164	linc01232	tissue	up	qRT-PCR		OS		32814086
165	PCAT6	tissue	up	qRT-PCR		OS		32825947
166	PMSB8-AS1	tissue	up	qRT-PCR		OS		32891166

a. AUC: area under the curve; b. DFS: disease-free survival; c. OS: overall survival; d. PFS: progression-free survival; "+" means that the lncRNA could be used as a prognostic biomarker in PC.
